# Mental Health Prevention and Promotion—A Narrative Review

**DOI:** 10.3389/fpsyt.2022.898009

**Published:** 2022-07-26

**Authors:** Vijender Singh, Akash Kumar, Snehil Gupta

**Affiliations:** Department of Psychiatry, All India Institute of Medical Sciences Bhopal, Bhopal, India

**Keywords:** mental health, promotion, prevention, protection, intervention, review, preventive psychiatry, novel interventions

## Abstract

Extant literature has established the effectiveness of various mental health promotion and prevention strategies, including novel interventions. However, comprehensive literature encompassing all these aspects and challenges and opportunities in implementing such interventions in different settings is still lacking. Therefore, in the current review, we aimed to synthesize existing literature on various mental health promotion and prevention interventions and their effectiveness. Additionally, we intend to highlight various novel approaches to mental health care and their implications across different resource settings and provide future directions. The review highlights the (1) concept of preventive psychiatry, including various mental health promotions and prevention approaches, (2) current level of evidence of various mental health preventive interventions, including the novel interventions, and (3) challenges and opportunities in implementing concepts of preventive psychiatry and related interventions across the settings. Although preventive psychiatry is a well-known concept, it is a poorly utilized public health strategy to address the population's mental health needs. It has wide-ranging implications for the wellbeing of society and individuals, including those suffering from chronic medical problems. The researchers and policymakers are increasingly realizing the potential of preventive psychiatry; however, its implementation is poor in low-resource settings. Utilizing novel interventions, such as mobile-and-internet-based interventions and blended and stepped-care models of care can address the vast mental health need of the population. Additionally, it provides mental health services in a less-stigmatizing and easily accessible, and flexible manner. Furthermore, employing decision support systems/algorithms for patient management and personalized care and utilizing the digital platform for the non-specialists' training in mental health care are valuable additions to the existing mental health support system. However, more research concerning this is required worldwide, especially in the low-and-middle-income countries.

## Introduction

Mental disorder has been recognized as a significant public health concern and one of the leading causes of disability worldwide, particularly with the loss of productive years of the sufferer's life (1). The Global Burden of Disease report (2019) highlights an increase, from around 80 million to over 125 million, in the worldwide number of Disability-Adjusted Life Years (DALYs) attributable to mental disorders. With this surge, mental disorders have moved into the top 10 significant causes of DALYs worldwide over the last three decades (2). Furthermore, this data does not include substance use disorders (SUDs), which, if included, would increase the estimated burden manifolds. Moreover, if the caregiver-related burden is accounted for, this figure would be much higher. Individual, social, cultural, political, and economic issues are critical mental wellbeing determinants. An increasing burden of mental diseases can, in turn, contribute to deterioration in physical health and poorer social and economic growth of a country (3). Mental health expenditure is roughly 3–4% of their Gross Domestic Products (GDPs) in developed regions of the world; however, the figure is abysmally low in low-and-middle-income countries (LMICs) (4). Untreated mental health and behavioral problems in childhood and adolescents, in particular, have profound long-term social and economic adverse consequences, including increased contact with the criminal justice system, lower employment rate and lesser wages among those employed, and interpersonal difficulties (5–8).

### Need for Mental Health (MH) Prevention

Longitudinal studies suggest that individuals with a lower level of positive wellbeing are more likely to acquire mental illness (9). Conversely, factors that promote positive wellbeing and resilience among individuals are critical in preventing mental illnesses and better outcomes among those with mental illness (10, 11). For example, in patients with depressive disorders, higher premorbid resilience is associated with earlier responses (12). On the contrary, patients with bipolar affective- and recurrent depressive disorders who have a lower premorbid quality of life are at higher risk of relapses (13).

Recently there has been an increased emphasis on the need to promote wellbeing and positive mental health in preventing the development of mental disorders, for poor mental health has significant social and economic implications (14–16). Research also suggests that mental health promotion and preventative measures are cost-effective in preventing or reducing mental illness-related morbidity, both at the society and individual level (17).

Although the World Health Organization (WHO) defines health as “a state of complete physical, mental, and social wellbeing and not merely an absence of disease or infirmity,” there has been little effort at the global level or stagnation in implementing effective mental health services (18). Moreover, when it comes to the research on mental health (vis-a-viz physical health), promotive and preventive mental health aspects have received less attention vis-a-viz physical health. Instead, greater emphasis has been given to the illness aspect, such as research on psychopathology, mental disorders, and treatment (19, 20). Often, physicians and psychiatrists are unfamiliar with various concepts, approaches, and interventions directed toward mental health promotion and prevention (11, 21).

Prevention and promotion of mental health are essential, notably in reducing the growing magnitude of mental illnesses. However, while health promotion and disease prevention are universally regarded concepts in public health, their strategic application for mental health promotion and prevention are often elusive. Furthermore, given the evidence of substantial links between psychological and physical health, the non-incorporation of preventive mental health services is deplorable and has serious ramifications. Therefore, policymakers and health practitioners must be sensitized about linkages between mental- and physical health to effectively implement various mental health promotive and preventive interventions, including in individuals with chronic physical illnesses (18).

The magnitude of the mental health problems can be gauged by the fact that about 10–20% of young individuals worldwide experience depression (22). As described above, poor mental health during childhood is associated with adverse health (e.g., substance use and abuse), social (e.g., delinquency), academic (e.g., school failure), and economic (high risk of poverty) adverse outcomes in adulthood (23). Childhood and adolescence are critical periods for setting the ground for physical growth and mental wellbeing (22). Therefore, interventions promoting positive psychology empower youth with the life skills and opportunities to reach their full potential and cope with life's challenges. Comprehensive mental health interventions involving families, schools, and communities have resulted in positive physical and psychological health outcomes. However, the data is limited to high-income countries (HICs) (24–28).

In contrast, in low and middle-income countries (LMICs) that bear the greatest brunt of mental health problems, including massive, coupled with a high treatment gap, such interventions remained neglected in public health (29, 30). This issue warrants prompt attention, particularly when global development strategies such as Millennium Development Goals (MDGs) realize the importance of mental health (31). Furthermore, studies have consistently reported that people with socioeconomic disadvantages are at a higher risk of mental illness and associated adverse outcomes; partly, it is attributed to the inequitable distribution of mental health services (32–35).

### Scope of Mental Health Promotion and Prevention in the Current Situation

Literature provides considerable evidence on the effectiveness of various preventive mental health interventions targeting risk and protective factors for various mental illnesses (18, 36–42). There is also modest evidence of the effectiveness of programs focusing on early identification and intervention for severe mental diseases (e.g., schizophrenia and psychotic illness, and bipolar affective disorders) as well as common mental disorders (e.g., anxiety, depression, stress-related disorders) (43–46). These preventive measures have also been evaluated for their cost-effectiveness with promising findings. In addition, novel interventions such as digital-based interventions and novel therapies (e.g., adventure therapy, community pharmacy program, and Home-based Nurse family partnership program) to address the mental health problems have yielded positive results. Likewise, data is emerging from LMICs, showing at least moderate evidence of mental health promotion intervention effectiveness. However, most of the available literature and intervention is restricted mainly to the HICs (47). Therefore, their replicability in LMICs needs to be established and, also, there is a need to develop locally suited interventions.

Fortunately, there has been considerable progress in preventive psychiatry over recent decades, including research on it. In the light of these advances, there is an accelerated interest among researchers, clinicians, governments, and policymakers to harness the potentialities of the preventive strategies to improve the availability, accessibility, and utility of such services for the community.

### The Concept of Preventive Psychiatry

#### Origins of Preventive Psychiatry

The history of preventive psychiatry can be traced back to the early 1900's with the foundation of the national mental health association (erstwhile mental health association), the committee on mental hygiene in New York, and the mental health hygiene movement (48). The latter emphasized the need for physicians to develop empathy and recognize and treat mental illness early, leading to greater awareness about mental health prevention (49). Despite that, preventive psychiatry remained an alien concept for many, including mental health professionals, particularly when the etiology of most psychiatric disorders was either unknown or poorly understood. However, recent advances in our understanding of the phenomena underlying psychiatric disorders and availability of the neuroimaging and electrophysiological techniques concerning mental illness and its prognosis has again brought the preventive psychiatry in the forefront (1).

#### Levels of Prevention

The literal meaning of “prevention” is “the act of preventing something from happening” (50); the entity being prevented can range from the risk factors of the development of the illness, the onset of illness, or the recurrence of the illness or associated disability. The concept of prevention emerged primarily from infectious diseases; measures like mass vaccination and sanitation promotion have helped prevent the development of the diseases and subsequent fatalities. The original preventive model proposed by the Commission on Chronic Illness in 1957 included primary, secondary, and tertiary preventions (48).

#### The Concept of Primary, Secondary, and Tertiary Prevention

**Figure 1:**
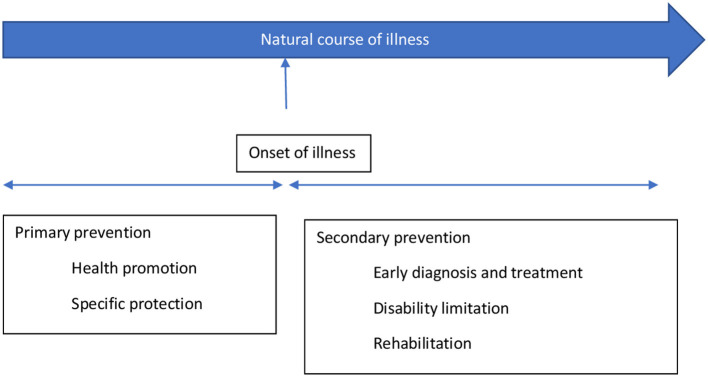
The concept of primary and secondary prevention [adopted from prevention: Primary, Secondary, Tertiary by Bauman et al. (51)].

The stages of prevention target distinct aspects of the illness's natural course; the primary prevention acts at the stage of pre-pathogenesis, that is, when the disease is yet to occur, whereas the secondary and tertiary prevention target the phase after the onset of the disease (51). Primary prevention includes health promotion and specific protection, while secondary and tertairy preventions include early diagnosis and treatment and measures to decrease disability and rehabilitation, respectively (51) (Figure 1).

The primary prevention targets those individuals vulnerable to developing mental disorders and their consequences because of their bio-psycho-social attributes. Therefore, it can be viewed as an intervention to prevent an illness, thereby preventing mental health morbidity and potential social and economic adversities. The preventive strategies under it usually target the general population or individuals at risk. Secondary and tertiary prevention targets those who have already developed the illness, aiming to reduce impairment and morbidity as soon as possible. However, these measures usually occur in a person who has already developed an illness, therefore facing related suffering, hence may not always be successful in curing or managing the illness. Thus, secondary and tertiary prevention measures target the already exposed or diagnosed individuals.

#### The Concept of Universal, Selective, and Indicated Prevention

The classification of health prevention based on primary/secondary/tertiary prevention is limited in being highly centered on the etiology of the illness; it does not consider the interaction between underlying etiology and risk factors of an illness. Gordon proposed another model of prevention that focuses on the degree of risk an individual is at, and accordingly, the intensity of intervention is determined. He has classified it into universal, selective, and indicated prevention. A universal preventive strategy targets the whole population irrespective of individual risk (e.g., maintaining healthy, psychoactive substance-free lifestyles); selective prevention is targeted to those at a higher risk than the general population (socio-economically disadvantaged population, e.g., migrants, a victim of a disaster, destitute, etc.). The indicated prevention aims at those who have established risk factors and are at a high risk of getting the disease (e.g., family history of psychiatric illness, history of substance use, certain personality types, etc.). Nevertheless, on the other hand, these two classifications (the primary, secondary, and tertiary prevention; and universal, selective, and indicated prevention) have been intended for and are more appropriate for physical illnesses with a clear etiology or risk factors (48).

**Figure 2:**
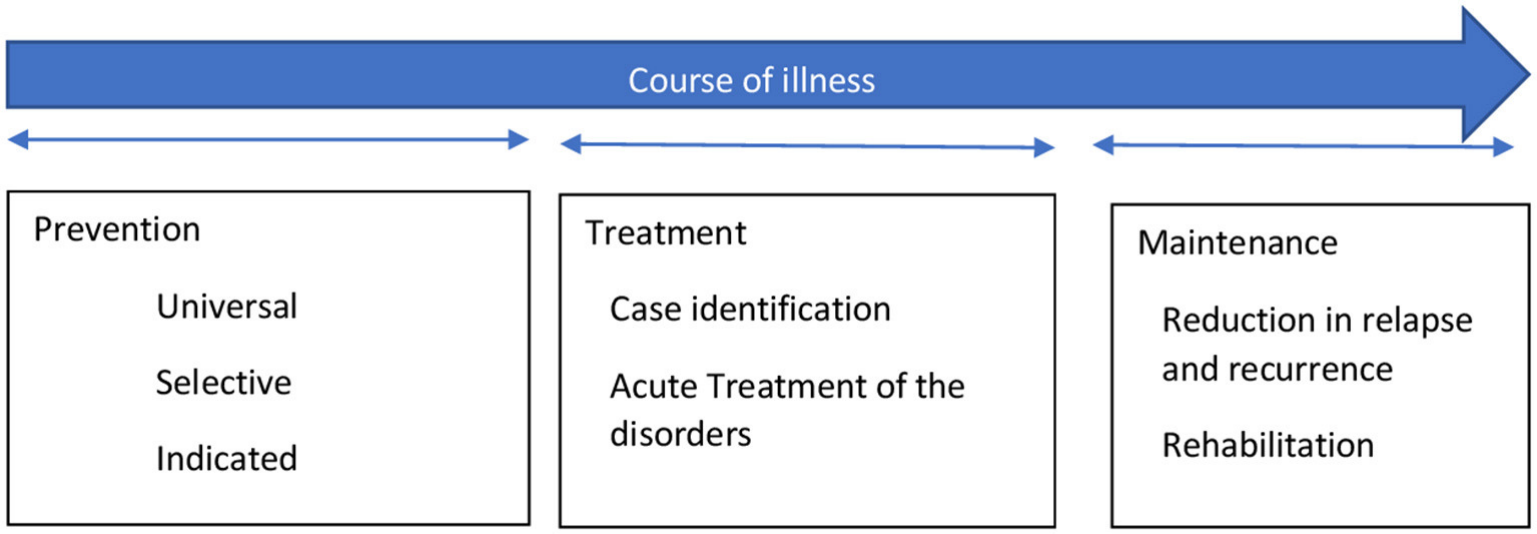
The interventions for mental illness as classified by the Institute of Medicine (IOM) Committee on Prevention of Mental Disorders [adopted from Mrazek and Haggerty (48)].

In 1994, the Institute of Medicine (IOM) Committee on Prevention of Mental Disorders proposed a new paradigm that classified primary preventive measures for mental illnesses into three categories. These are indicated, selected, and universal preventive interventions (refer Figure 2). According to this paradigm, primary prevention was limited to interventions done before the onset of the mental illness (48). In contrast, secondary and tertiary prevention encompasses treatment and maintenance measures (Figure 2).

Although the boundaries between prevention and treatment are often more overlapping than being exclusive, the new paradigm can be used to avoid confusion stemming from the common belief that prevention can take place at all parts of mental health management (48). The onset of mental illnesses can be prevented by risk reduction interventions, which can involve reducing risk factors in an individual and strengthening protective elements in them. It aims to target modifiable factors, both risk, and protective factors, associated with the development of the illness through various general and specific interventions. These interventions can work across the lifespan. The benefits are not restricted to reduction or delay in the onset of illness but also in terms of severity or duration of illness (48).On the spectrum of mental health interventions, universal preventive interventions are directed at the whole population without identifiable risk factors. The interventions are beneficial for the general population or sub-groups. Prenatal care and childhood vaccination are examples of preventative measures that have benefited both physical and mental health. Selective preventive mental health interventions are directed at people or a subgroup with a significantly higher risk of developing mental disorders than the general population. Risk groups are those who, because of their vulnerabilities, are at higher risk of developing mental illnesses, e.g., infants with low-birth-weight (LBW), vulnerable children with learning difficulties or victims of maltreatment, elderlies, etc. Specific interventions are home visits and new-born day care facilities for LBW infants, preschool programs for all children living in resource-deprived areas, support groups for vulnerable elderlies, etc. Indicated preventive interventions focus on high-risk individuals who have developed minor but observable signs or symptoms of mental disorder or genetic risk factors for mental illness. However, they have not fulfilled the criteria of a diagnosable mental disorder. For instance, the parent-child interaction training program is an indicated prevention strategy that offers support to children whose parents have recognized them as having behavioral difficulties.

The overall objective of mental health promotion and prevention is to reduce the incidence of new cases, additionally delaying the emergence of mental illness. However, promotion and prevention in mental health complement each other rather than being mutually exclusive. Moreover, combining these two within the overall public health framework reduces stigma, increases cost-effectiveness, and provides multiple positive outcomes (18).

### How Prevention in Psychiatry Differs From Other Medical Disorders

Compared to physical illnesses, diagnosing a mental illness is more challenging, particularly when there is still a lack of objective assessment methods, including diagnostic tools and biomarkers. Therefore, the diagnosis of mental disorders is heavily influenced by the assessors' theoretical perspectives and subjectivity. Moreover, mental illnesses can still be considered despite an individual not fulfilling the proper diagnostic criteria led down in classificatory systems, but there is detectable dysfunction. Furthermore, the precise timing of disorder initiation or transition from subclinical to clinical condition is often uncertain and inconclusive (48). Therefore, prevention strategies are well-delineated and clear in the case of physical disorders while it's still less prevalent in mental health parlance.

### Terms, Definitions, and Concepts

The terms mental health, health promotion, and prevention have been differently defined and interpreted. It is further complicated by overlapping boundaries of the concept of promotion and prevention. Some commonly used terms in mental health prevention have been tabulated (Table 1) (18).

**Table 1:** Commonly used terms in mental health prevention.

**Term**	**Common definitions**
Mental health	WHO defines MH as a state of wellbeing in which a person is cognizant of their potential, equipped to deal with typical life stressors, capable of productive and fruitful employment, and capable of contributing to their community (18).
Mental health promotion	It is a means of empowering people to take more control of their own health and wellbeing. It encompasses several initiatives aimed at positive effects on mental health and relates to mental wellbeing rather than mental illness (18). Any intervention is done to improve individuals' and communities' mental health and wellbeing (52). Improving an individual's, family, group's, or community's ability to reinforce or promote good emotional, cognitive, and associated experiences (53).
Mental health protection	There is no universally agreed-upon definition of mental health protection. The definition has been derived from the literal meaning of protection, that states “the act of keeping somebody/something safe so that he/she is not harmed or damaged.” In the prevention model of illness, health protection comes under primary prevention to prevent the occurrence of the illness, physical or mental.

### Mental Health Promotion and Protection

The term “mental health promotion” also has definitional challenges as it signifies different things to different individuals. For some, it means the treatment of mental illness; for others, it means preventing the occurrence of mental illness; while for others, it means increasing the ability to manage frustration, stress, and difficulties by strengthening one's resilience and coping abilities (54). It involves promoting the value of mental health and improving the coping capacities of individuals rather than amelioration of symptoms and deficits.

Mental health promotion is a broad concept that encompasses the entire population, and it advocates for a strengths-based approach and tries to address the broader determinants of mental health. The objective is to eliminate health inequalities *via* empowerment, collaboration, and participation. There is mounting evidence that mental health promotion interventions improve mental health, lower the risk of developing mental disorders (48, 55, 56) and have socioeconomic benefits (24). In addition, it strives to increase an individual's capacity for psychosocial wellbeing and adversity adaptation (11).

However, the concepts of mental health promotion, protection, and prevention are intrinsically linked and intertwined. Furthermore, most mental diseases result from complex interaction risk and protective factors instead of a definite etiology. Facilitating the development and timely attainment of developmental milestones across an individual's lifespan is critical for positive mental health (57). Although mental health promotion and prevention are essential aspects of public health with wide-ranging benefits, their feasibility and implementation are marred by financial and resource constraints. The lack of cost-effectiveness studies, particularly from the LMICs, further restricts its full realization (47, 58, 59).

Despite the significance of the topic and a considerable amount of literature on it, a comprehensive review is still lacking that would cover the concept of mental health promotion and prevention and simultaneously discusses various interventions, including the novel techniques delivered across the lifespan, in different settings, and level of prevention. Therefore, this review aims to analyze the existing literature on various mental health promotion and prevention-based interventions and their effectiveness. Furthermore, its attempts to highlight the implications of such intervention in low-resource settings and provides future directions. Such literature would add to the existing literature on mental health promotion and prevention research and provide key insights into the effectiveness of such interventions and their feasibility and replicability in various settings.

## Methodology

For the current review, key terms like “mental health promotion,” OR “protection,” OR “prevention,” OR “mitigation” were used to search relevant literature on Google Scholar, PubMed, and Cochrane library databases, considering a time period between 2000 to 2019 (Supplementary Material 1). However, we have restricted our search till 2019 for non-original articles (reviews, commentaries, viewpoints, etc.), assuming that it would also cover most of the original articles published until then. Additionally, we included original papers from the last 5 years (2016–2021) so that they do not get missed out if not covered under any published review. The time restriction of 2019 for non-original articles was applied to exclude papers published during the Coronavirus disease (COVID-19) pandemic as the latter was a significant event, bringing about substantial change and hence, it warranted a different approach to cater to the MH needs of the population, including MH prevention measures. Moreover, the COVID-19 pandemic resulted in the flooding of novel interventions for mental health prevention and promotion, specifically targeting the pandemic and its consequences, which, if included, could have biased the findings of the current review on various MH promotion and prevention interventions.

A time frame of about 20 years was taken to see the effectiveness of various MH promotion and protection interventions as it would take substantial time to be appreciated in real-world situations. Therefore, the current paper has put greater reliance on the review articles published during the last two decades, assuming that it would cover most of the original articles published until then.

The above search yielded 320 records: 225 articles from Google scholar, 59 articles from PubMed, and 36 articles from the Cochrane database flow-diagram of records screening. All the records were title/abstract screened by all the authors to establish the suitability of those records for the current review; a bibliographic- and gray literature search was also performed. In case of any doubts or differences in opinion, it was resolved by mutual discussion. Only those articles directly related to mental health promotion, primary prevention, and related interventions were included in the current review. In contrast, records that discussed any specific conditions/disorders (post-traumatic stress disorders, suicide, depression, etc.), specific intervention (e.g., specific suicide prevention intervention) that too for a particular population (e.g., disaster victims) lack generalizability in terms of mental health promotion or prevention, those not available in the English language, and whose full text was unavailable were excluded. The findings of the review were described narratively.

## Findings

### Interventions for Mental Health Promotion and Prevention and Their Evidence

Various interventions have been designed for mental health promotion and prevention. They are delivered and evaluated across the regions (high-income countries to low-resource settings, including disaster-affiliated regions of the world), settings (community-based, school-based, family-based, or individualized); utilized different psychological constructs and therapies (cognitive behavioral therapy, behavioral interventions, coping skills training, interpersonal therapies, general health education, etc.); and delivered by different professionals/facilitators (school-teachers, mental health professionals or paraprofessionals, peers, etc.). The details of the studies, interventions used, and outcomes have been provided in Supplementary Table 1. Below we provide the synthesized findings of the available research.

The majority of the available studies were quantitative and experimental. Randomized controlled trials comprised a sizeable proportion of the studies; others were quasi-experimental studies and, a few, qualitative studies. The studies primarily focussed on school students or the younger population, while others were explicitly concerned with the mental health of young females (60). Newer data is emerging on mental health promotion and prevention interventions for elderlies (e.g., dementia) (61). The majority of the research had taken a broad approach to mental health promotion (62). However, some studies have focused on universal prevention (63, 64) or selective prevention (65–68). For instance, the Resourceful Adolescent Program (RAPA) was implemented across the schools and has utilized cognitive-behavioral and interpersonal therapies and reported a significant improvement in depressive symptoms. Some of the interventions were directed at enhancing an individual's characteristics like resilience, behavior regulation, and coping skills (ZIPPY's Friends) (69), while others have focused on the promotion of social and emotional competencies among the school children and attempted to reduce the gap in such competencies across the socio-economic classes (“Up” program) (70) or utilized expressive abilities of the war-affected children (Writing for Recover (WfR) intervention) (71) to bring about an improvement in their psychological problems (a type of selective prevention) (62) or harnessing the potential of Art, in the community-based intervention, to improve self-efficacy, thus preventing mental disorders (MAD about Art program) (72). Yet, others have focused on strengthening family (60, 73), community relationships (62), and targeting modifiable risk factors across the life course to prevent dementia among the elderlies and also to support the carers of such patients (61).

Furthermore, more of the studies were conducted and evaluated in the developed parts of the world, while emerging economies, as anticipated, far lagged in such interventions or related research. The interventions that are specifically adapted for local resources, such as school-based programs involving paraprofessionals and teachers in the delivery of mental health interventions, were shown to be more effective (62, 74). Likewise, tailored approaches for low-resource settings such as LMICs may also be more effective (63). Some of these studies also highlight the beneficial role of a multi-dimensional approach (68, 75) and interventions targeting early lifespan (76, 77).

### Newer Insights: How to Harness Digital Technology and Novel Methods of MH Promotion and Protection

With the advent of digital technology and simultaneous traction on mental health promotion and prevention interventions, preventive psychiatrists and public health experts have developed novel techniques to deliver mental health promotive and preventive interventions. These encompass different settings (e.g., school, home, workplace, the community at large, etc.) and levels of prevention (universal, selective, indicated) (78–80).

The advanced technologies and novel interventions have broadened the scope of MH promotion and prevention, such as addressing the mental health issues of individuals with chronic medical illness (81, 82), severe mental disorders (83), children and adolescents with mental health problems, and geriatric population (78). Further, it has increased the accessibility and acceptability of such interventions in a non-stigmatizing and tailored manner. Moreover, they can be integrated into the routine life of the individuals.

For instance, Internet-and Mobile-based interventions (IMIs) have been utilized to monitor health behavior as a form of MH prevention and a stand-alone self-help intervention. Moreover, the blended approach has expanded the scope of MH promotive and preventive interventions such as face-to-face interventions coupled with remote therapies. Simultaneously, it has given way to the stepped-care (step down or step-up care) approach of treatment and its continuation (79). Also, being more interactive and engaging is particularly useful for the youth.

The blended model of care has utilized IMIs to a varying degree and at various stages of the psychological interventions. This includes IMIs as a supplementary approach to the face-to-face-interventions (FTFI), FTFI augmented by behavior intervention technologies (BITs), BITs augmented by remote human support, and fully automated BITs (84).

The stepped care model of mental health promotion and prevention strategies includes a stepped-up approach, wherein BITs are utilized to manage the prodromal symptoms, thereby preventing the onset of the full-blown episode. In the Stepped-down approach, the more intensive treatments (in-patient or out-patient based interventions) are followed and supplemented with the BITs to prevent relapse of the mental illness, such as for previously admitted patients with depression or substance use disorders (85, 86).

Similarly, the latest research has developed newer interventions for strengthening the psychological resilience of the public or at-risk individuals, which can be delivered at the level of the home, such as, e.g., nurse family partnership program (to provide support to the young and vulnerable mothers and prevent childhood maltreatment) (87); family healing together program aimed at improving the mental health of the family members living with persons with mental illness (PwMI) (88). In addition, various novel interventions for MH promotion and prevention have been highlighted in the Table 2.

**Table 2:** Depiction of various novel mental health promotion and prevention strategies.

**Name of program/intervention and MH condition**	**Setting and modality**	**Component of the program**	**Condition**	**Remarks**
**Level of prevention: Health promotion**				
Community-Based MH Services Community pharmacy program (Australia)	*Mode of delivery:* physical *Resource person:* community pharmacist who dispense medicines to the public	• Distributing in-store leaflets on mental wellbeing, posters display and linking with existing national • MH organizations/ campaigns	MH promotion of adults visitors to the pharmacy.	• A suitable environment for MH promotion, particularly for a person with lived experience. • Community pharmacy is widely distributed and easily accessible. • Lack of privacy and the busy pharmacy environment were, however, identified as potential barriers.
Technology-based mental health promotional intervention for later life (89)	*Study design:* Systematic review	Technology use for elderly education, computer/internet exposure or training, telephone/internet communication, and computer gaming.	*n* = 25 interventional studies, significant positive effects on psychosocial outcomes among the intervention recipients.	• Digital inclusion and training of elderlies are important. • Initiatives early in the life can promote and protect wellbeing in later life.
**GUIDE**- training of teachers in MH promotion (Canada) (90)	*Study design:* Multisite pre-post study	• Duration of in-class teaching: 8–12 h, 1 day of teachers training. • Teacher's self-study guide, teacher's knowledge self-assessment, student evaluation materials, and six-core modules for the teachers^#^. Supplementary Materials: A-Vs and web-linked resources.	*Results:* Significant improvements in teachers' knowledge and attitudes toward mental wellbeing and illness with large effect sizes.	A scalable model can be incorporated in the routine professional training and education for the teachers.
**Myhealth** Magazine (Canada) (90) MH literacy	Online interactive health and MH programming and materials for teachers and students on MH literacy	• Series of online and classroom-based activities and workshops. • Smartphone and desktop/ tablet versions also available	• *Findings*: a high percentage of students use these resources for MH information. • Students with considerable distress use more online resources and likely to access further help (e.g., school-based MH center) • High satisf'n with web site	A scalable model that has high usability and accessibility.
**Level of prevention: Universal prevention**				
Community program/campaign R U OK? (2009, Australia) And Beyondblue campaign for the public (91, 92)	• *Mode of delivery:* online/ telephonic conversion. • Condition: Suicide prevention	• To connect with those experiencing MH problems. Providing resources and tips for the same. • People are advised to ask; listen non-judgementally; encourage the person to take action, e.g., visit an MHP; and follow up with that person.	Knowledge about the causes and recognition of mental illness had increased over time, increased willingness of the people to talk with others about their MH problems and seek professional help, including decreasing stigma a/w help-seeking.	Can be replicated in the low-resource setting; however, feasibility and effectiveness studies are warranted before implementation.
Workplace	• Workplace wellness program (Canada) • Mode of delivery: offline and online activities	Promoted MH as well as healthy behaviors such as physical activity, adequate sleep, proper nutrition, and work-life balance to encourage presenteeism	Increased presentism, decreasing workplace stress and depression.	• The program needs to be tailored to the needs which could vary from place to place. • Implementation in low-resource settings may be a challenge.
	• Green exercise (Norway) (93) Municipality employees • Condition: workplace stress	Stress Mgt. program: exercising in nature (information meeting and 2 exercise sessions, biking bout and circuit strengthening exercise), over traditional indoor exercise routines, in promoting MH and reducing stress.	Higher environmental potential for restoration and Positive Affect, which persisted on 10 wks follow-up.	• May be logistically challenging. • Require further exploration.
	• Guided E-Learning for Managers • *Mode of delivery:* online	Intervention to identify sources of stress, better understand the link of mental and physical illness and improve managers' capacity to help their employees proactively deal with stressful working conditions	Better understanding among the managers further impacts the psychosocial needs of their teams.	• Lesser engagement of the managers. • Greater involvement is required. • Identifying key personnel challenging.
• School-based program secondary education students (age 13–16 yrs.) (93) • Condition: eating disorders	*Mode of delivery:* Young[E]spirit stepped program (IA) vs. online-psychoeducation intervention (CG)	Screening and customized risk feedback with recommendations for specific self-help modules, monitoring of symptoms and risk behavior and synchronous group and Individual online chats till the individual FTF counseling.	• *n* = 1,667 adolescent receiving the online intervention (IA) in two waves. • *Outcome:* Prevention of EDs • *Results:* significantly reduced ED onset rates in the IA vs. CG) schools in the first wave (5.6%, vs. 9.6%) but no significant diff. in the second wave	Replicability, acceptability, and feasibility concerns in low-resource settings.
**Level of prevention: Selective prevention**				
• Home-based • Nurse family partnership program (Elmira, Memphis, and Denver) (87). • Condition: Women with some psycho'cal problems due to early pregnancy (<19 yrs), single mother, unmarried women low-socio-economic status, etc.	• *Study design: a* review of 3RCTs • *IA:* women receive home visitation services during pregnancy and in the first 2 yrs post-partum • *CG:* comparison services.	• Specific assessments of maternal, child, and family functioning that correspond to pregnancy and 2 yrs thereafter. • Dietary monitoring, assessment and mgt. of smoking, alcohol, and other illicit substance use; teach women to identify the signs and Symptoms of pregnancy complications; curricula are used to promote parent-child interaction.	• *n* = 1,139. • improved the quality of diets, lesser cigarette smoking, fewer preterm delivery, fewer behavioral problems due to substance use, • IA: Children more communicative and responsive toward their mothers, had lesser emergency visits, lesser childhood maltreatment, fewer behavioral problems.	• Reduce stigma among mothers with psychological problems. • Can be replicated in a country like India with a huge community health workforce (Anganwadi workers, ANM, etc.)
Family healing together program	• Family mental health recovery program. • *Mode of delivery:* Online	Eight-week online aimed at recovery-oriented psychoeducation and coping with an MH challenge in the family.	• *Study design:* Qualitative. • Emphasized hope toward recovery, improved accessibility. • The curriculum was user friendly incorporating diversity to make it useful for everyone. • Greater need of such programs Need of scholarship and sponsorship for participation • The service fee is a limitation.	Replication in resource-poor and LMIC can be an issue.
• (SHUTi) (Australia) (94) • *Condition:* sleep problems in patients with a history of depression	• Mode of delivery: online • Unguided fully automated Internet-based intervention for (SHUTi) or to Healthwatch.	• Six sequential modules comprising Sleep hygiene, cognitive restructuring, relapse prevention, • Maintenance of sleep diary • *Outcome:* PHQ-9	• *N* = SHUTi (*n* = 574) or HealthWatch (*n* = 575). • Significant improvement in complaints of insomnia and depression symptom at 6 wks and 6 months FUs (vs. Healthwatch gr.). • Decrease in prevention of the depressive episose non-significant	Long-term data is warranted to conclude its efficacy in the prevention of depressive episodes.
Internet chat groups for relapse prevention (95) • Conditions: various mental illnesses	• Transdiagnostic non-manualized Internet-chat group as a stepped-care intervention following in-patient psychotherapy. • Mode of delivery: online	• *Group activities:* 8–10 participants/gr., who communicate with a therapist in an internet chat room @ once/week at a fixed time for 1 ½ h to communicate in written format. • Number of sessions:10–12 • *The focus of chat:* support patients in maintaining treatment gains and assisting them in practicing skills they learned during their hospital stay to everyday life.	• *N* = 152, • *IA:* internet chat groups • *CG:* TAU • Outcome: 1 year after discharge. • For any relapse: fewer participants (22.2%) of IA (vs. CG: 46.5%) experienced a relapse	Generalizability across the setting and users' privacy could be the issues.
**Level of prevention: Indicated prevention**				
• Get.ON mood enhancer prevention (96) • Condition: sub-syndromal depression	• Internet-based cognitive-behavioral intervention (IA) vs. online passive psychoeducation intervention (CG). • *Mode of delivery:* online	• Involves behavior therapy and problem-solving therapy. • Total six lessons with two sessions/week, • Lessons involve text, exercises, and testimonials which are interactive involving Audio (relaxation ex.)-Visual clips (concept of behavioral activation). Transfer of tasks (home assignments) in daily routine.	• *N* = 406, • Significantly lesser participants of the IA (32 vs. 47% CG) experienced an MDD at 12 m follow-up. • NNT = 5.9	The utility needs to be established in those with previous depressive episodes.
• Internet-based CBT (97) • Condition: self-report symptoms of depressive, but not meeting the diagnostic criteria for MDD	• Internet-based CBT (Delivered in comic form) vs. waitlist. • Comic format increases the motivation of the participants and facilitated easy learning.	• Six- web-based training in stress mgt. delivered over 6 weeks with each session of 30 min/week. • *Components:* self-monitoring, cognitive restructuring, assertiveness, problem-solving, and relaxation with homework	• *N* = 822 • lower incidence of the depressive episode at the 12 months FU, with the prevalence of 0.8 and 3.9% in IA and CG, respectively. • NTT = 32	Needs to be tailored as per the different cultural contexts.
• Project UPLIFT (98) • Condition: adult epilepsy patients with • Sub-syndromic depression	• 8-week web or telephone-delivered mindfulness-based • stand-alone intervention vs. TAU waitlist (CG)	• 8-module, delivered in a group format. • Component: increase knowledge about depression; observing, challenging, and changing of thoughts; relaxing and coping techniques; attention and mindfulness; focusing on pleasure; the significance of reinforcement; and relapse prevention. • *Outcome:* self-reported outcomes on depression and MDD, knowledge/skills, and life satisfaction. • At baseline, 10 weeks, and 20 weeks FUs.	• *N* = 64 • *Result:* incidence of depressive episode and depressive symptoms were significantly lower IA vs. CG. No difference b/w web-based vs. telephonic intervention. • Better knowledge, skills and life satisfaction increased significantly in the IA.	• Increased accessibility for persons with epilepsy whose mobility has been affected by the illness. • Could cater to the hard-to-reach population. • Can be replicated in other disabling medical illnesses.
• Naslund et al. (99) • Digital Technology for Building Capacity of Non-specialist Health Workers for Task-Sharing and Scaling Up Mental HealthCare Globally	• Type of article: • Perspective. • Role of digital technology for enabling non-specialist health professionals in implementing evidence-based MH interventions	• Use of digital platforms in different LMICs for providing training to HCWs, diagnosis and treating mental disorders and providing an integrated service. Such as: • The Atmiyata Intervention and The SMART MH Project in India, • TACTS for Thinking Healthy Program in Pakistan, • The Friendship Bench in Zimbabwe, • The Allillanchu Project in Peru, • Community-based LEAN in China, • EXPONATE for Perinatal Depression in Nigeria	Some of the interventions have reported significant positive outcomes while other interventions are being evaluated for their effectiveness	These interventions highlight the potential of better implementation of task sharing with non-specialist health professional approach and may help in reducing the global treatment gap esp. in low resource countries
• Maron et al. (100) • Manifesto for an international digital mental health network	• The international network for digital mental health (IDMHN): work for implementation of digital technologies in MH services like DocuMental: a clinical decision support system (DSS) for MH service staff including physician, nurses, health care managers and coordinators • i-PROACH: a cloud based intelligent platform for research, outcome, assessment and care in mental health utilizing DSS, algorithm on generic data, digital phenotyping, and artificial intelligence	• Diagnostic module: digitized structured ICD-10 diagnostic criteria liked with DSS algorithms for increased accuracy and allow verification and differentiation. • Treatment module: linked to DSS algorithms for medication and treatment plan selection which can help in planning treatment in a standardized manner and to avoid mistreatment • History and routine assessment modules: for comprehensive and standardized assessments	Such novel interventions/algorithm have potential to address the current mental health needs especially by making it more transparent, personalized, standardized, more proactive and responsive for collaboration with other specialties and organizations.	This type of model may be best suited for HICs at the same time implementation in LMICs need to be assessed
• Antonova et al. (101) • Coping With COVID-19: Mindfulness-Based Approaches for Mitigating Mental Health Crisis	Type of article - Viewpoint	Various interventions that have utilized mindfulness skills like observing, non-judging, non-reacting, acting with awareness, and describing such as NHS's Mind app, Headspace (teaching meditation *via* a website or a phone application)	Help healthcare personnel to cope with excessive anxiety, panic, and exhaustion while fulfilling their duties and responsibilities during the COVID-19 pandemic	Such novel interventions based on the mindfulness practices can help individuals to cope with the difficulties posed by major life events such as pandemic.

# *MH stigma, understanding MH and wellness, information about different mental disorders, experiences of mental illnesses, help seeking and garnering support, and importance of positive MH*.

*a/w, associated with; A-V, audio-visual; b/w, between; CBT, Cognitive Behavioral Therapy; CES-Dep., Center for Epidemiologic Studies-Depression scale; CG, control group; FU, follow-up; GAD, generalized anxiety disorders-7; IA, intervention arm; HCWs, Health Care Workers; LMIC, low and middle-income countries; MDD, major depressive disorders; mgt, management; MH, mental health; MHP, mental health professional; MINI, mini neuropsychiatric interview; NNT, number needed to treat; PHQ-9, patient health questionnaire; TAU, treatment as usual*.

Furthermore, school/educational institutes-based interventions such as school-Mental Health Magazines to increase mental health literacy among the teachers and students have been developed (80). In addition, workplace mental health promotional activities have targeted the administrators, e.g., guided “e-learning” for the managers that have shown to decrease the mental health problems of the employees (102).

Likewise, digital technologies have also been harnessed in strengthening community mental health promotive/preventive services, such as the mental health first aid (MHFA) Books on Prescription initiative in New Zealand provided information and self-help tools through library networks and trained book “prescribers,” particularly in rural and remote areas (103).

Apart from the common mental disorders such as depression, anxiety, and behavioral disorders in the childhood/adolescents, novel interventions have been utilized to prevent the development of or management of medical, including preventing premature mortality and psychological issues among the individuals with severe mental illnesses (SMIs), e.g., Lets' talk about tobacco-web based intervention and motivational interviewing to prevent tobacco use, weight reduction measures, and promotion of healthy lifestyles (exercise, sleep, and balanced diets) through individualized devices, thereby reducing the risk of cardiovascular disorders (83). Similarly, efforts have been made to improve such individuals' coping skills and employment chances through the WorkingWell mobile application in the US (104).

Apart from the digital-based interventions, newer, non-digital-based interventions have also been utilized to promote mental health and prevent mental disorders among individuals with chronic medical conditions. One such approach in adventure therapy aims to support and strengthen the multi-dimensional aspects of self. It includes the physical, emotional or cognitive, social, spiritual, psychological, or developmental rehabilitation of the children and adolescents with cancer. Moreover, it is delivered in the natural environment outside the hospital premises, shifting the focus from the illness model to the wellness model (81). Another strength of this intervention is it can be delivered by the nurses and facilitate peer support and teamwork.

Another novel approach to MH prevention is gut-microbiota and dietary interventions. Such interventions have been explored with promising results for the early developmental disorders (Attention deficit hyperactive disorder, Autism spectrum disorders, etc.) (105). It works under the framework of the shared vulnerability model for common mental disorders and other non-communicable diseases and harnesses the neuroplasticity potential of the developing brain. Dietary and lifestyle modifications have been recommended for major depressive disorders by the Clinical Practice Guidelines in Australia (106). As most childhood mental and physical disorders are determined at the level of the *in-utero* and early after the birth period, targeting maternal nutrition is another vital strategy. The utility has been expanded from maternal nutrition to women of childbearing age. The various novel mental health promotion and prevention strategies are shown in Table 2.

Newer research is emerging that has utilized the digital platform for training non-specialists in diagnosis and managing individuals with mental health problems, such as Atmiyata Intervention and The SMART MH Project in India, and The Allillanchu Project in Peru, to name a few (99). Such frameworks facilitate task-sharing by the non-specialist and help in reducing the treatment gap in these countries. Likewise, digital algorithms or decision support systems have been developed to make mental health services more transparent, personalized, outcome-driven, collaborative, and integrative; one such example is DocuMental, a clinical decision support system (DSS). Similarly, frameworks like i-PROACH, a cloud-based intelligent platform for research outcome assessment and care in mental health, have expanded the scope of the mental health support system, including promoting research in mental health (100). In addition, COVID-19 pandemic has resulted in wider dissemination of the applications based on the evidence-based psycho-social interventions such as National Health Service's (NHS's) Mind app and Headspace (teaching meditation *via* a website or a phone application) that have utilized mindfulness-based practices to address the psychological problems of the population (101).

### Challenges in Implementing Novel MH Promotion and Prevention Strategies

Although novel interventions, particularly internet and mobile-based interventions (IMIs), are effective models for MH promotion and prevention, their cost-effectiveness requires further exploration. Moreover, their feasibility and acceptability in LMICs could be challenging. Some of these could be attributed to poor digital literacy, digital/network-related limitations, privacy issues, and society's preparedness to implement these interventions.

These interventions need to be customized and adapted according to local needs and context, for which implementation and evaluative research are warranted. In addition, the infusion of more human and financial resources for such activities is required. Some reports highlight that many of these interventions do not align with the preferences and use the pattern of the service utilizers. For instance, one explorative research on mental health app-based interventions targeting youth found that despite the burgeoning applications, they are not aligned with the youth's media preferences and learning patterns. They are less interactive, have fewer audio-visual displays, are not youth-specific, are less dynamic, and are a single touch app (107).

Furthermore, such novel interventions usually come with high costs. In low-resource settings where service utilizers have limited finances, their willingness to use such services may be doubtful. Moreover, insurance companies, including those in high-income countries (HICs), may not be willing to fund such novel interventions, which restricts the accessibility and availability of interventions.

Research points to the feasibility and effectiveness of incorporating such novel interventions in routine services such as school, community, primary care, or settings, e.g., in low-resource settings, the resource persons like teachers, community health workers, and primary care physicians are already overburdened. Therefore, their willingness to take up additional tasks may raise skepticism. Moreover, the attitudinal barrier to moving from the traditional service delivery model to the novel methods may also impede.

Considering the low MH budget and less priority on the MH prevention and promotion activities in most low-resource settings, the uptake of such interventions in the public health framework may be lesser despite the latter's proven high cost-effectiveness. In contrast, policymakers may be more inclined to invest in the therapeutic aspects of MH.

### Road Ahead

Such interventions open avenues for personalized and precision medicine/health care vs. the traditional model of MH promotion and preventive interventions (108, 109). For instance, multivariate prediction algorithms with methods of machine learning and incorporating biological research, such as genetics, may help in devising tailored, particularly for selected and indicated prevention, interventions for depression, suicide, relapse prevention, etc. (79). Therefore, more research in this area is warranted.

To be more clinically relevant, greater biological research in MH prevention is required to identify those at higher risk of developing given mental disorders due to the existing risk factors/prominent stress (110). For instance, researchers have utilized the transcriptional approach to identify a biological fingerprint for susceptibility (denoting abnormal early stress response) to develop post-traumatic stress disorders among the psychological trauma survivors by analyzing the expression of the Peripheral blood mononuclear cell gene expression profiles (111). Identifying such biological markers would help target at-risk individuals through tailored and intensive interventions as a form of selected prevention.

Similarly, such novel interventions can help in targeting the underlying risk such as substance use, poor stress management, family history, personality traits, etc. and protective factors, e.g., positive coping techniques, social support, resilience, etc., that influences the given MH outcome (79). Therefore, again, it opens the scope of tailored interventions rather than a one-size-fits-all model of selective and indicated prevention for various MH conditions.

Furthermore, such interventions can be more accessible for the hard-to-reach populations and those with significant mental health stigma. Finally, they play a huge role in ensuring the continuity of care, particularly when community-based MH services are either limited or not available. For instance, IMIs can maintain the improvement of symptoms among individuals previously managed in-patient, such as for suicide, SUDs, etc., or receive intensive treatment like cognitive behavior therapy (CBT) for depression or anxiety, thereby helping relapse prevention (86, 112). Hence, such modules need to be developed and tested in low-resource settings.

IMIs (and other novel interventions) being less stigmatizing and easily accessible, provide a platform to engage individuals with chronic medical problems, e.g., epilepsy, cancer, cardiovascular diseases, etc., and non-mental health professionals, thereby making it more relevant and appealing for them.

Lastly, research on prevention-interventions needs to be more robust to adjust for the pre-intervention matching, high attrition rate, studying the characteristics of treatment completers vs. dropouts, and utilizing the intention-to-treat analysis to gauge the effect of such novel interventions (78).

## Recommendations for Low-and-Middle-Income Countries

Although there is growing research on the effectiveness and utility of mental health promotion/prevention interventions across the lifespan and settings, low-resource settings suffer from specific limitations that restrict the full realization of such public health strategies, including implementing the novel intervention. To overcome these challenges, some of the potential solutions/recommendations are as follows:

- The mental health literacy of the population should be enhanced through information, education, and communication (IEC) activities. In addition, these activities should reduce stigma related to mental problems, early identification, and help-seeking for mental health-related issues.
- Involving teachers, workplace managers, community leaders, non-mental health professionals, and allied health staff in mental health promotion and prevention is crucial.
- Mental health concepts and related promotion and prevention should be incorporated into the education curriculum, particularly at the medical undergraduate level.
- Training non-specialists such as community health workers on mental health-related issues across an individual's life course and intervening would be an effective strategy.
- Collaborating with specialists from other disciplines, including complementary and alternative medicines, would be crucial. A provision of an integrated health system would help in increasing awareness, early identification, and prompt intervention for at-risk individuals.
- Low-resource settings need to develop mental health promotion interventions such as community-and school-based interventions, as these would be more culturally relevant, acceptable, and scalable.
- Utilizing a digital platform for scaling mental health services (e.g., telepsychiatry services to at-risk populations) and training the key individuals in the community would be a cost-effective framework that must be explored.
- Infusion of higher financial and human resources in this area would be a critical step, as, without adequate resources, research, service development, and implementation would be challenging.
- It would also be helpful to identify vulnerable populations and intervene in them to prevent the development of clinical psychiatric disorders.
- Lastly, involving individuals with lived experiences at the level of mental health planning, intervention development, and delivery would be cost-effective.

## Conclusion

Clinicians, researchers, public health experts, and policymakers have increasingly realized mental health promotion and prevention. Investment in Preventive psychiatry appears to be essential considering the substantial burden of mental and neurological disorders and the significant treatment gap. Literature suggests that MH promotive and preventive interventions are feasible and effective across the lifespan and settings. Moreover, various novel interventions (e.g., internet-and mobile-based interventions, new therapies) have been developed worldwide and proven effective for mental health promotion and prevention; such interventions are limited mainly to HICs.

Despite the significance of preventive psychiatry in the current world and having a wide-ranging implication for the wellbeing of society and individuals, including those suffering from chronic medical problems, it is a poorly utilized public health field to address the population's mental health needs. Lately, researchers and policymakers have realized the untapped potentialities of preventive psychiatry. However, its implementation in low-resource settings is still in infancy and marred by several challenges. The utilization of novel interventions, such as digital-based interventions, and blended and stepped-care models of care, can address the enormous mental health need of the population. Additionally, it provides mental health services in a less-stigmatizing and easily accessible, and flexible manner. More research concerning this is required from the LMICs.

## Author Contributions

VS, AK, and SG: methodology, literature search, manuscript preparation, and manuscript review. All authors contributed to the article and approved the submitted version.

## Conflict of Interest

The authors declare that the research was conducted in the absence of any commercial or financial relationships that could be construed as a potential conflict of interest.

## Publisher's Note

All claims expressed in this article are solely those of the authors and do not necessarily represent those of their affiliated organizations, or those of the publisher, the editors and the reviewers. Any product that may be evaluated in this article, or claim that may be made by its manufacturer, is not guaranteed or endorsed by the publisher.

## References

[1] Trivedi JK Tripathi A Dhanasekaran S Moussaoui D. Preventive psychiatry: concept appraisal and future directions. Int J Soc Psychiatry. (2014) 60:321–9. 10.1177/0020764013488570 23788436

[2] Ng M Fleming T Robinson M Thomson B Graetz N Margono C. Global, regional, and national prevalence of overweight and obesity in children and adults during 1980–2013: a systematic analysis for the Global Burden of Disease Study 2013. Lancet Lond Engl. (2014) 384:766–81. 10.1016/S0140-6736(14)60460-8 24880830 PMC4624264

[3] Allen J Balfour R Bell R Marmot M. Social determinants of mental health. Int Rev Psychiatry Abingdon Engl. (2014) 26:392–407. 10.3109/09540261.2014.928270 25137105

[4] Organization IL. Mental Health in the Workplace. Introduction. Geneva: International Labour Organization (2000). Available online at: https://public.ebookcentral.proquest.com/choice/publicfullrecord.aspx?p=4954519 (accessed March 2, 2022).

[5] Scott S Knapp M Henderson J Maughan B. Financial cost of social exclusion: follow up study of antisocial children into adulthood. BMJ. (2001) 323:191. 10.1136/bmj.323.7306.191 11473907 PMC35269

[6] Chen H Cohen P Kasen S Johnson JG Berenson K Gordon K. Impact of adolescent mental disorders and physical illnesses on quality of life 17 years later. Arch Pediatr Adolesc Med. (2006) 160:93–9. 10.1001/archpedi.160.1.93 16389217

[7] McCrone P Knapp M Fombonne E. The Maudsley long-term follow-up of child and adolescent depression. Predicting costs in adulthood. Eur Child Adolesc Psychiatry. (2005) 14:407–13. 10.1007/s00787-005-0491-6 16254770

[8] Fergusson DM Horwood LJ Ridder EM. Show me the child at seven: the consequences of conduct problems in childhood for psychosocial functioning in adulthood. J Child Psychol Psychiatry. (2005) 46:837–49. 10.1111/j.1469-7610.2004.00387.x 16033632

[9] Wood AM Joseph S. The absence of positive psychological (eudemonic) well-being as a risk factor for depression: a ten year cohort study. J Affect Disord. (2010) 122:213–7. 10.1016/j.jad.2009.06.032 19706357

[10] Burton NW Pakenham KI Brown WJ. Feasibility and effectiveness of psychosocial resilience training: a pilot study of the READY program. Psychol Health Med. (2010) 15:266–77. 10.1080/13548501003758710 20480432

[11] Kalra G Christodoulou G Jenkins R Tsipas V Christodoulou N Lecic-Tosevski D. Mental health promotion: guidance and strategies. Eur Psychiatry J Assoc Eur Psychiatr. (2012) 27:81–6. 10.1016/j.eurpsy.2011.10.001 22197146

[12] Min JA Lee NB Lee CU Lee C Chae JH. Low trait anxiety, high resilience, and their interaction as possible predictors for treatment response in patients with depression. J Affect Disord. (2012) 137:61–9. 10.1016/j.jad.2011.12.026 22244377

[13] Thunedborg K Black CH Bech P. Beyond the Hamilton depression scores in long-term treatment of manic-melancholic patients: prediction of recurrence of depression by quality of life measurements. Psychother Psychosom. (1995) 64:131–40. 10.1159/000289002 8657843

[14] Lopez AD Mathers CD Ezzati M Jamison DT Murray CJ. Global Burden of Disease Risk Factors. Washington, DC: World Bank (2006). Available online at: http://www.ncbi.nlm.nih.gov/books/NBK11812/ (accessed February 28, 2022).

[15] World Health Organization. WHO European Ministerial Conference on Mental Health (1st : 2005 : Helsinki F, World Health Organization. Regional Office for Europe. Mental health action plan for Europe : facing the challenges, building solutions. In: First WHO European Ministerial Conference on Mental Health, Helsinki, Finland. (EUR/04/5047810/7). (2005). Available from: https://apps.who.int/iris/handle/10665/107627 (accessed January 12–15, 2005). 16250587

[16] Green Paper. Improving the Mental Health of the Population Towards a Strategy on Mental Health for the European Union. Brussels. p. 30.

[17] Cuijpers P Van Straten A Smit F. Preventing the incidence of new cases of mental disorders: a meta-analytic review. J Nerv Ment Dis. (2005) 193:119–25. 10.1097/01.nmd.0000152810.76190.a6 15684914

[18] Saxena S Maulik PK World Health Organization. Prevention and Promotion in Mental Health. Geneva: World Health Organization (2002).

[19] Herrman H. The need for mental health promotion. Aust N Z J Psychiatry. (2001) 35:709–15. 10.1046/j.1440-1614.2001.00947.x 11990880

[20] Ryff CD Singer B. Psychological well-being: meaning, measurement, and implications for psychotherapy research. Psychother Psychosom. (1996) 65:14–23. 10.1159/000289026 8838692

[21] Monshat K Herrman H. What does “mental health promotion” mean to psychiatry trainees? Australas Psychiatry Bull R Aust N Z Coll Psychiatr. (2010) 18:589. 10.3109/10398562.2010.500330 21117853

[22] Kieling C Baker-Henningham H Belfer M Conti G Ertem I Omigbodun O. Child and adolescent mental health worldwide: evidence for action. Lancet Lond Engl. (2011) 378:1515–25. 10.1016/S0140-6736(11)60827-1 22008427

[23] Jenkins R Baingana F Ahmad R McDaid D Atun R. Social, economic, human rights and political challenges to global mental health. Ment Health Fam Med. (2011) 8:87–96. 22654971 PMC3178190

[24] Jané-Llopis E Barry M Hosman C Patel V. Mental health promotion works: a review. Promot Educ. (2005) 12(Suppl.):9–25. 10.1177/10253823050120020103x 15966248

[25] Nores M Barnett WS. Benefits of early childhood interventions across the world: (Under) Investing in the very young. Econ Educ Rev. (2010) 29:271–82. 10.1016/j.econedurev.2009.09.001

[26] Baker-Henningham H López Bóo F. Early Childhood Stimulation Interventions in Developing Countries: A Comprehensive Literature Review. Bonn: Institute for the Study of Labor. (2010). 10.2139/ssrn.1700451 Available online at: https://papers.ssrn.com/sol3/papers.cfm?abstract_id=1700451

[27] Stewart-Brown SL Schrader-McMillan A. Parenting for mental health: what does the evidence say we need to do? Report of Workpackage 2 of the DataPrev project. Health Promot Int. (2011) 26(Suppl.1):i10–28. 10.1093/heapro/dar056 22079931

[28] Weare K Nind M. Mental health promotion and problem prevention in schools: what does the evidence say? Health Promot Int. (2011) 26(Suppl.1):i29–69. 10.1093/heapro/dar075 22079935

[29] Barry MM Clarke AM Jenkins R Patel V. A systematic review of the effectiveness of mental health promotion interventions for young people in low and middle income countries. BMC Public Health. (2013) 13:1–19. 10.1186/1471-2458-13-835 24025155 PMC3848687

[30] Patel V Flisher AJ Nikapota A Malhotra S. Promoting child and adolescent mental health in low and middle income countries. J Child Psychol Psychiatry. (2008) 49:313–34. 10.1111/j.1469-7610.2007.01824.x 18093112

[31] Miranda JJ Patel V. Achieving the millennium development goals: does mental health play a role? PLoS Med. (2005) 2:e291. 10.1371/journal.pmed.0020291 16156692 PMC1201694

[32] Fryers T Melzer D Jenkins R. Social inequalities and the common mental disorders: a systematic review of the evidence. Soc Psychiatry Psychiatr Epidemiol. (2003) 38:229–37. 10.1007/s00127-003-0627-2 12719837

[33] Jenkins R Bhugra D Bebbington P Brugha T Farrell M Coid J. Debt, income and mental disorder in the general population. Psychol Med. (2008) 38:1485–93. 10.1017/S0033291707002516 18184442

[34] Lund C Breen A Flisher AJ Kakuma R Corrigall J Joska JA. Poverty and common mental disorders in low and middle income countries: a systematic review. Soc Sci Med. (2010) 71:517–28. 10.1016/j.socscimed.2010.04.027 20621748 PMC4991761

[35] Blas E Kurup AS. Equity, Social Determinants and Public Health Programmes. Available online at: https://apps.who.int/iris/handle/10665/44289 (accessed March 2, 2022).

[36] Durlak JA Wells AM. Primary prevention mental health programs: the future is exciting. Am J Community Psychol. (1997) 25:233–43. 10.1023/A:1024674631189 9226868

[37] Durlak JA. Primary prevention mental health programs for children and adolescents are effective. J Ment Health. (1998) 7:463–9. 10.1080/0963823981784235101116

[38] Lecic-Tosevski D Christodoulou G Herrman H Hosman C Jenkins R Newton J. WPA consensus statement on psychiatric prevention. Dyn Psychiatr Dyn Psychiatry. (2003) 36:307–19.8908416

[39] Kolbe LJ. Meta-analysis of interventions to prevent mental health problems among youth: a public health commentary. Am J Community Psychol. (1997) 25:227–32. 10.1023/A:1024622614351 9226867

[40] Olds DL Eckenrode J Henderson CR Kitzman H Powers J Cole R. Long-term effects of home visitation on maternal life course and child abuse and neglect. Fifteen-year follow-up of a randomized trial. J Am Med Assoc. (1997) 278:637–43. 10.1001/jama.1997.03550080047038 9272895

[41] Andrews G Wilkinson DD. The prevention of mental disorders in young people. Med J Aust. (2002) 177:S97–100. 10.5694/j.1326-5377.2002.tb04865.x 12358565

[42] HEALTHEVIDENCE. The Prevention of Mental Disorders in School-Aged Children: Current State of the Field. Available online at: https://www.healthevidence.org/view-article.aspx?a=prevention-mental-disorders-school-aged-children-current-state-field-15455 (accessed March 2, 2022).

[43] Marshall M Rathbone J. Early intervention for psychosis. Cochrane Database Syst Rev. (2011) 6:CD004718. 10.1002/14651858.CD004718.pub3 21678345 PMC4163966

[44] Cuijpers P van Straten A Smits N Smit F. Screening and early psychological intervention for depression in schools : systematic review and meta-analysis. Eur Child Adolesc Psychiatry. (2006) 15:300–7. 10.1007/s00787-006-0537-4 16572276

[45] Neil AL Christensen H. Australian school-based prevention and early intervention programs for anxiety and depression: a systematic review. Med J Aust. (2007) 186:305–8. 10.5694/j.1326-5377.2007.tb00906.x 17371212

[46] Merry S McDowell H Hetrick S Bir J Muller N. Psychological and/or educational interventions for the prevention of depression in children and adolescents. Cochrane Database Syst Rev. (2004) 1:CD003380. 10.1002/14651858.CD003380.pub2 14974014

[47] Zechmeister I Kilian R McDaid D. Is it worth investing in mental health promotion and prevention of mental illness? A systematic review of the evidence from economic evaluations. BMC Public Health. (2008) 8:1–11. 10.1186/1471-2458-8-20 18211677 PMC2245925

[48] Mrazek PJ Haggerty RJ. Institute of Medicine (US) Committee on Prevention of Mental Disorders. Reducing Risks for Mental Disorders: Frontiers for Preventive Intervention Research. Washington, DC: National Academies Press (US) (1994). Available online at: http://www.ncbi.nlm.nih.gov/books/NBK236319/ (accessed March 2, 2022). 25144015

[49] Meyer A. The mental hygiene movement. Can Med Assoc J. (1918) 8:632–4. 20311133 PMC1585211

[50] Cambridge. Prevention Meaning in the Cambridge English Dictionary. (2022). Available online at: https://dictionary.cambridge.org/dictionary/english/prevention (accessed March 2, 2022).

[51] Baumann LC Karel A. Prevention: primary, secondary, tertiary. In: Gellman MD, Turner JR, editors, Encyclopedia of Behavioral Medicine. New York, NY: Springer (2013). p. 1532–4. Available online at: 10.1007/978-1-4419-1005-9_135 (accessed March 2, 2022).

[52] Commonwealth Department of Health and Aged Care. Introduction. In: Rickwood D. editor. Promotion, Prevention and Early Intervention for Mental Health – A Monograph. Canberra: Mental Health and Special Programs Branch, Commonwealth Department of Health and Aged Care (2000). p. 1–8.

[53] Hodgson R Abbasi T Clarkson J. Effective mental health promotion: a literature review. Health Educ J. (1996) 55:55–74. 10.1177/001789699605500106

[54] Sartorius N. Health promotion strategies: keynote address. Can J Public Health Rev Can Santee Publique. (1988) 79:S3–5. 3242787

[55] Jané-Llopis E Hosman C Jenkins R Anderson P. Predictors of efficacy in depression prevention programmes. Meta-analysis. Br J Psychiatry J Ment Sci. (2003) 183:384–97. 10.1192/bjp.183.5.384 14594912

[56] World Health Organization. Prevention of Mental Disorders: Effective Interventions and Policy Options: Summary Report [cited 2022 Mar 5] (2004).

[57] Min JA Lee CU Lee C. Mental health promotion and illness prevention: a challenge for psychiatrists. Psychiatry Investig. (2013) 10:307. 10.4306/pi.2013.10.4.307 24474978 PMC3902147

[58] Drummond MF Sculpher MJ Torrance GW O'Brien BJ Stoddart GL. Methods for the Economic Evaluation of Health Care Programmes. Oxford University Press (2005). Available online at: https://econpapers.repec.org/bookchap/oxpobooks/9780198529453.htm (accessed March 5, 2022).

[59] Wanless D. Securing Good Health for the Whole Population: Population Health Trends. London: HM Treasury (2003). p. 51.

[60] Brady M Assaad R Ibrahim B Salem A Salem R Zibani N. Providing New Opportunities to Adolescent Girls in Socially Conservative Settings: The Ishraq Program in Rural Upper Egypt—Full Report. Poverty Gend Youth. (2007) Available online at: https://knowledgecommons.popcouncil.org/departments_sbsr-pgy/226 (accessed March 15, 2022).

[61] The Lancet Commission on Dementia Prevention Intervention Care: a call for action. Irish Journal of Psychological Medicine. Cambridge Core. (2021). Available online at: https://www.cambridge.org/core/journals/irish-journal-of-psychological-medicine/article/lancet-commission-on-dementia-prevention-intervention-and-care-a-call-for-action/3E7ED3B3B08161D9FC2B75FD8268703D (accessed May 21, 2022). 10.1017/ipm.2018.4 31187723

[62] Balaji M Andrews T Andrew G Patel V. The acceptability, feasibility, and effectiveness of a population-based intervention to promote youth health: an exploratory study in Goa, India. J Adolesc Health Off Publ Soc Adolesc Med. (2011) 48:453–60. 10.1016/j.jadohealth.2010.07.029 21501803 PMC4991743

[63] Rivet-Duval E Heriot S Hunt C. Preventing adolescent depression in mauritius: a universal school-based program. Child Adolesc Ment Health. (2011) 16:86–91. 10.1111/j.1475-3588.2010.00584.x 32847217

[64] Holen S Waaktaar T Lervåg A Ystgaard M. The effectiveness of a universal school-based programme on coping and mental health: a randomised, controlled study of Zippy's Friends. Educ Psychol. (2012) 32:657–77. 10.1080/01443410.2012.686152

[65] Loughry M Ager A Flouri E Khamis V Afana AH Qouta S. The impact of structured activities among Palestinian children in a time of conflict. J Child Psychol Psychiatry. (2006) 47:1211–8. 10.1111/j.1469-7610.2006.01656.x 17176376

[66] Qouta SR Palosaari E Diab M Punamäki RL. Intervention effectiveness among war-affected children: a cluster randomized controlled trial on improving mental health. J Trauma Stress. (2012) 25:288–98. 10.1002/jts.21707 22648703

[67] Karam EG Fayyad J Nasser Karam A Cordahi Tabet C Melhem N Mneimneh Z. Effectiveness and specificity of a classroom-based group intervention in children and adolescents exposed to war in Lebanon. World Psychiatry Off J World Psychiatr Assoc WPA. (2008) 7:103–9. 10.1002/j.2051-5545.2008.tb00170.x 18560511 PMC2430518

[68] Jewkes R Nduna M Levin J Jama N Dunkle K Puren A. Impact of Stepping Stones on incidence of HIV and HSV-2 and sexual behaviour in rural South Africa: cluster randomised controlled trial. BMJ. (2008) 337:a506. 10.1136/bmj.a506 18687720 PMC2505093

[69] Mishara BL Ystgaard M. Effectiveness of a mental health promotion program to improve coping skills in young children: Zippy's Friends. Early Child Res Q. (2006) 21:110–23. 10.1016/j.ecresq.2006.01.002

[70] Nielsen L Meilstrup C Nelausen MK Koushede V Holstein BE. Promotion of social and emotional competence: Experiences from a mental health intervention applying a whole school approach. Health Educ. (2015) 115:339–56. 10.1108/HE-03-2014-0039

[71] Lange-Nielsen II Kolltveit S Thabet AAM Dyregrov A Pallesen S Johnsen TB. Short-term effects of a writing intervention among adolescents in Gaza. J Loss Trauma. (2012) 17:403–22. 10.1080/15325024.2011.650128

[72] Mueller J Alie C Jonas B Brown E Sherr L. A quasi-experimental evaluation of a community-based art therapy intervention exploring the psychosocial health of children affected by HIV in South Africa. Trop Med Int Health TM IH. (2011) 16:57–66. 10.1111/j.1365-3156.2010.02682.x 21073640

[73] Bell CC Bhana A Petersen I McKay MM Gibbons R Bannon W. Building protective factors to offset sexually risky behaviors among black youths. J Natl Med Assoc. (2008) 100:936–44. 10.1016/S0027-9684(15)31408-5 18717144 PMC2536567

[74] Bonhauser M Fernandez G Püschel K Yañez F Montero J Thompson B. Improving physical fitness and emotional well-being in adolescents of low socioeconomic status in Chile: results of a school-based controlled trial. Health Promot Int. (2005) 20:113–22. 10.1093/heapro/dah603 15788528

[75] Ager A Akesson B Stark L Flouri E Okot B McCollister F. The impact of the school-based Psychosocial Structured Activities (PSSA) program on conflict-affected children in northern Uganda. J Child Psychol Psychiatry. (2011) 52:1124–33. 10.1111/j.1469-7610.2011.02407.x 21615734

[76] Caldarella P Christensen L Kramer TJ Kronmiller K. Promoting social and emotional learning in second grade students: a study of the strong start curriculum. Early Child Educ J. (2009) 37:51–6. 10.1007/s10643-009-0321-4

[77] Yamamoto T Matsumoto Y Bernard ME. Effects of the cognitive-behavioral you can do it! Education program on the resilience of Japanese elementary school students: a preliminary investigation. Int J Educ Res. (2017) 86:50–8. 10.1016/j.ijer.2017.08.006

[78] Neumann L Dapp U Jacobsen W van Lenthe F von Renteln-Kruse W. The MINDMAP project: mental well-being in urban environments. Z Gerontol Geriatr. (2017) 50:588–602. 10.1007/s00391-017-1290-7 28819693 PMC5649390

[79] Ebert DD Cuijpers P Muñoz RF Baumeister H. Prevention of mental health disorders using internet- and mobile-based interventions: a narrative review and recommendations for future research. Front Psychiatry. (2017) 8:116. 10.3389/fpsyt.2017.00116 28848454 PMC5554359

[80] Sharma A Sharma SD Sharma M. Mental health promotion: a narrative review of emerging trends. Curr Opin Psychiatry. (2017) 30:339–45. 10.1097/YCO.0000000000000347 28661906

[81] Epstein I. Adventure therapy: a mental health promotion strategy in pediatric oncology. J Pediatr Oncol Nurs. (2004) 21:103–10. 10.1177/1043454203262684 15125554

[82] Natale P Palmer SC Ruospo M Saglimbene VM Rabindranath KS Strippoli GF. Psychosocial interventions for preventing and treating depression in dialysis patients. Cochrane Database Syst Rev. (2019) 2019:CD004542. 10.1002/14651858.CD004542.pub3 31789430 PMC6886341

[83] Naslund JA Aschbrenner KA. Digital technology for health promotion: opportunities to address excess mortality in persons living with severe mental disorders. Evid Based Ment Health. (2019) 22:17–22. 10.1136/ebmental-2018-300034 30559332 PMC6359972

[84] Ebert D Erbe D. Internet-basierte psychologische interventionen. In: Berking M Rief W editors, Klinische Psychologie und Psychotherapie für Bachelor. Berlin; Heidelberg: Springer (2012). p. 131–40. 10.1007/978-3-642-25523-6_12

[85] Golkaramnay V Bauer S Haug S Wolf M Kordy H. The exploration of the effectiveness of group therapy through an Internet chat as aftercare: a controlled naturalistic study. Psychother Psychosom. (2007) 76:219–25. 10.1159/000101500 17570960

[86] Kok G Bockting C Burger H Smit F Riper H. Mobile Cognitive Therapy: adherence and acceptability of an online intervention in remitted recurrently depressed patients. Internet Interv. (2014) 1:65–73. 10.1016/j.invent.2014.05.002

[87] Olds DL Hill PL O'Brien R Racine D Moritz P. Taking preventive intervention to scale: the nurse-family partnership. Cogn Behav Pract. (2003) 10:278–90. 10.1016/S1077-7229(03)80046-9

[88] Estrada S. Families healing together: exploring a family recovery online course. Qual Rep. (2016) 21:1216–1231. 10.46743/2160-3715/2016.2181

[89] Forsman A Nordmyr J Park A Matosevic T McDaid D Wahlbeck K. Technology-based interventions for mental health promotion in later life: an evidence review: Anna Forsman. Eur J Public Health. (2016) 26(Suppl.1):ckw172.044. 10.1093/eurpub/ckw172.044

[90] Kutcher S Bagnell A Wei Y. Mental health literacy in secondary schools: a Canadian approach. Child Adolesc Psychiatr Clin N Am. (2015) 24:233–44. 10.1016/j.chc.2014.11.007 25773321

[91] Mok K Donovan R Hocking B Maher B Lewis R Pirkis J. Stimulating community action for suicide prevention: findings on the effectiveness of the Australian R U OK? Campaign Int J Ment Health Promot. (2016) 18:213–21. 10.1080/14623730.2016.1209423

[92] Jorm AF Christensen H Griffiths KM. The impact of beyondblue: the national depression initiative on the Australian public's recognition of depression and beliefs about treatments. Aust N Z J Psychiatry. (2005) 39:248–54. 10.1080/j.1440-1614.2005.01561.x 15777361

[93] Lindenberg K Kordy H. Wirksamkeit eines gestuften, Internetvermittelten Ansatzes zur Prävention von Essstörungen bei Schülern der 7. bis 10. Klasse Kindh Entwickl. (2015) 24:55–63. 10.1026/0942-5403/a000152

[94] Christensen H Batterham PJ Gosling JA Ritterband LM Griffiths KM Thorndike FP. Effectiveness of an online insomnia program (SHUTi) for prevention of depressive episodes (the GoodNight Study): a randomised controlled trial. Lancet Psychiatry. (2016) 3:333–41. 10.1016/S2215-0366(15)00536-2 26827250

[95] Bauer S Wolf M Haug S Kordy H. The effectiveness of internet chat groups in relapse prevention after inpatient psychotherapy. Psychother Res J Soc Psychother Res. (2011) 21:219–26. 10.1080/10503307.2010.547530 21347978

[96] Buntrock C Ebert DD Lehr D Cuijpers P Riper H Smit F. Evaluating the efficacy and cost-effectiveness of web-based indicated prevention of major depression: design of a randomised controlled trial. BMC Psychiatry. (2014) 14:25. 10.1186/1471-244X-14-25 24485283 PMC3914724

[97] Imamura K Kawakami N Furukawa TA Matsuyama Y Shimazu A Umanodan R. Does Internet-based cognitive behavioral therapy (iCBT) prevent major depressive episode for workers? A 12-month follow-up of a randomized controlled trial. Psychol Med. (2015) 45:1907–17. 10.1017/S0033291714003006 25562115

[98] Thompson NJ Patel AH Selwa LM Stoll SC Begley CE Johnson EK. Expanding the efficacy of project UPLIFT: distance delivery of mindfulness-based depression prevention to people with epilepsy. J Consult Clin Psychol. (2015) 83:304–13. 10.1037/a0038404 25495361 PMC4380523

[99] Naslund JA Shidhaye R Patel V. Digital technology for building capacity of nonspecialist health workers for task sharing and scaling up mental health care globally. Harv Rev Psychiatry. (2019) 27:181–92. 10.1097/HRP.0000000000000217 30958400 PMC6517068

[100] Maron E Baldwin DS Balõtšev R Fabbri C Gaur V Hidalgo-Mazzei D. Manifesto for an international digital mental health network. Digit Psychiatry. (2019) 2:14–24. 10.1080/2575517X.2019.1617575

[101] Antonova E Schlosser K Pandey R Kumari V. Coping with COVID-19: mindfulness-based approaches for mitigating mental health crisis. Front Psychiatry. (2021) 2021:563417. 10.3389/fpsyt.2021.563417 33833695 PMC8021723

[102] Stansfeld SA Berney L Bhui K Chandola T Costelloe C Hounsome N. Pilot Study of a Randomised Trial of a Guided E-Learning Health Promotion Intervention for Managers Based on Management Standards for the Improvement of Employee Well-Being Reduction of Sickness Absence: The GEM (Guided E-learning for Managers) Study. Public Health Research. Southampton: NIHR Journals Library (2015). Available online at: http://www.ncbi.nlm.nih.gov/books/NBK310357/ (accessed February 22, 2022). 26269873

[103] Carty S Thompson L Berger S Jahnke K Llewellyn R. Books on Prescription - community-based health initiative to increase access to mental health treatment: an evaluation. Aust N Z J Public Health. (2016) 40:276–8. 10.1111/1753-6405.12507 27027774

[104] Nicholson J Carpenter-Song EA MacPherson LH Tauscher JS Burns TC Lord SE. Developing the WorkingWell mobile app to promote job tenure for individuals with serious mental illnesses. Psychiatr Rehabil J. (2017) 40:276–82. 10.1037/prj0000201 27322395 PMC7480984

[105] Dawson SL Dash SR Jacka FN. The importance of diet and gut health to the treatment and prevention of mental disorders. Int Rev Neurobiol. (2016) 131:325–46. 10.1016/bs.irn.2016.08.009 27793225

[106] Malhi GS Bassett D Boyce P Bryant R Fitzgerald PB Fritz K. Royal Australian and New Zealand College of Psychiatrists clinical practice guidelines for mood disorders. Aust N Z J Psychiatry. (2015) 49:1087–206. 10.1177/0004867415617657 26643054

[107] Michel T Slovak P Fitzpatrick G. An explorative review of youth mental health apps for prevention and promotion. In: Proceedings of the 13th EAI International Conference on Pervasive Computing Technologies for Healthcare - Demos and Posters. Trento: EAI (2019). Available online at: http://eudl.eu/doi/10.4108/eai.20-5-2019.2283578 (accessed February 16, 2022).

[108] King M Bottomley C Bellón-Saameño JA Torres-Gonzalez F Švab I Rifel J. An international risk prediction algorithm for the onset of generalized anxiety and panic syndromes in general practice attendees: predictA. Psychol Med. (2011) 41:1625–39. 10.1017/S0033291710002400 21208520

[109] Wang J Sareen J Patten S Bolton J Schmitz N Birney A. prediction algorithm for first onset of major depression in the general population: development and validation. J Epidemiol Community Health. (2014) 68:418–24. 10.1136/jech-2013-202845 24391206

[110] Insel TR Scolnick EM. Cure therapeutics and strategic prevention: raising the bar for mental health research. Mol Psychiatry. (2006) 11:11–7. 10.1038/sj.mp.4001777 16355250 PMC1586099

[111] Segman RH Shefi N Goltser-Dubner T Friedman N Kaminski N Shalev AY. Peripheral blood mononuclear cell gene expression profiles identify emergent post-traumatic stress disorder among trauma survivors. Mol Psychiatry. (2005) 10:500–13. 10.1038/sj.mp.4001636 15685253

[112] Bockting CL Kok GD Kamp L van der Smit F van Valen E Schoevers R. Disrupting the rhythm of depression using Mobile Cognitive Therapy for recurrent depression: randomized controlled trial design and protocol. BMC Psychiatry. (2011) 11:12. 10.1186/1471-244X-11-12 21235774 PMC3036590

