# Supplementary material for: Mental Health Prevention and Promotion—A Narrative Review
Source: Front Psychiatry. 2022 Jul 26;13:898009. doi: 10.3389/fpsyt.2022.898009 (PMC9360426; doi:10.3389/fpsyt.2022.898009)
Supplement: Supplementary file 1 [file Table_1.docx]

**Supplementary table 1. Depiction of various Mental Health Promotion and Prevention interventions and their effectiveness**

| **Author, publication year & place** | **Type of study*** | **Program** | **Study Design** | **Sample size** | **Type of prevention** | **Findings of the study** | **Remark** |
| --- | --- | --- | --- | --- | --- | --- | --- |
| Mishara, 2006 (1) (Denmark & Lithuania) | Quantitative | ZIPPY’s Friends  Effectiveness of MH promotion program to improve coping skills in young children | Non-randomized Experimental trial | Students Lithuania: IG: 314  CG: 104  Denmark:  IG: 322  CG: 110 | MH Promotion | - Increases ability to cope with everyday’ stress & negative events - Decreases problems that arise due to stressful situations - Development of effective adaptive coping skills | - Resilience & Coping skills development &behaviour self-regulation as key elements of MH promotion |
| Clarke, 2014 (2) Ireland | Quantitative | ZIPPY’s Friends  evaluating impact of a school-based emotional wellbeing program for disadvantaged school children | Cluster RCT | IG:544 students  CG: 222 students | MH Promotion | - Increases ability to cope with day to days stress & negative events - Decrease problems arising from stressful events - Promotes development of adaptive coping skills |  |
| Dufour 2011 (3) Canada (Quebec) | Quantitative | ZIPPY’s Friends  Improving Children’s adaptation: a School MH Promotion Program | Cluster RCT | IG: 310  (From 16 classes)  CG: 303  (From 19 classes) | MH Promotion | - Increase ability to cope with everyday life adversities & negative events - Decrease problems that arise from unfavourable situations - Developing of adaptive coping techniques |  |
| Holen 2012 (4)  Norway | Quantitative | ZIPPY’s Friends  The effectiveness of universal school-based program on coping & MH | RCT | IG: 686  (47 classes, 18 schools)  CG: 638  (44 classes, 17 schools) | MH Promotion | - Better ability to cope with everyday life adversities & negative events - Decrease problems that arise from stressful situations - Adaptive coping skills |  |
| Clark 2015 (5) Ireland | Quantitative | ZIPPY’s Friends  Implementation of emotional wellbeing program for primary school children | Participatory workshop using RCT | IG: 544  Students  CG: 222 students  Workshop | MH Promotion | - Increase ability to cope with everyday life adversities & negative events - Decrease problems that arise secondary to stressful events - Adaptive coping techniques |  |
| Malti, 2008 (6) (United States) | Mixed method | RALLY (Responsive Advocacy for Life and Learning in Youth) | Quasi-experimental | 92 students | MH Promotion | - Improved resilience, greater learning interest, & decrease risk-taking | - The research emphasizes the benefits of   effective relationships |
| Nielsen 2015 (7) Denmark | Quantitative | “Up”, promotion of social & emotional competence through multi-component interventions | Pre-post design | 589 students (2 school) | MH Promotion | - Enhancing social & emotional competencies to improve MH - Enhanced positivity of school MH environment | - Emphasizes that relationship & behaviour &empathy as key elements |
| Caldarella 2009 (8)  United States (Utah) | Quantitative | “Strong start”  promoting social & emotional learning in 2^nd^ grade students | Quasi-Experimental Non-Equivalent Control Group | 26 students | MH Promotion | - Prevent emotional & behavioral problems through promotion of social & emotional wellbeing | - Emphasizes effectiveness of MH promotion program implemented in the early lifespan |
| Yamamoto 2017 (9)  Tokyo | Quantitative | “You Can Do it! Education” Resilience of Japanese elementary school students | Quasi-Experimental Intervention, Control Group | IG: N=78, CG: N=47 | MH Promotion | - Efficacy in enhancing resilience in schools | - Emphasizes effectiveness of MH promotion program implemented in early in life |
| Srikala & Kumar, 2010 (10) India | Quantitative | Life Skills education programme | Quasi-Experimental- random selection of schools with matched control design | N= 1028 adolescents control received standard civic education classes | MH Promotion | - Significant improvement in: self-esteem, perceived self-efficacy, pro-social behaviour, & perceived adequate coping - Better adjustment in school | - Emphasizes on life skills intervention in middle adolescents (14-16 years) |
| Banhouser et al, 2005 (11) Santiago, Chile | Quantitative | School-based physical fitness programme | Quasi-experimental design | N= 198 students from high school. | Mental & Physical Health Promotion | - Significant improvement in adolescents’ anxiety & self-esteem scores - Significant increases in physical fitness: in terms of oxygen capacity, speed as well as jump performance scores | - Intervention was tailored to preferences of the students, expertise of the teachers & availability of the local resources. |
| Smith et al. 2008; Caldwell et al, 2010 (12,13) Cape Town South Africa | Quantitative | Health Wide Program | Quasi-experimental | N= 2193 adolescents mean age 14yrs. Life Orientation curriculum taught in control schools | MH Promotion | - Significant increase in intrinsic motivation - Decrease in introjected motivation - Increase in perception about availability of condoms in IG - Effects on alcohol & cigarette use were more for girls | - Program delivered by class teacher |
| De Villiers & van den Berg 2012 (14) South Africa | Quantitative | Resiliency Programme  Intervention providing 15 sessions on enhancing emotional regulation, stress management, interpersonal skills & effective problem solving | “Solomon Four Group Design” | N= 161 children aged 11-12yrs. from 4 schools Waitlist control, 3months follow up | MH Promotion | - Improvement observed in terms of interpersonal strength, emotional regulation, self-appraisal, & emotional reactivity - Improved self-appraisal scores at three months FU | - Taps the potential of the early adolescence to develop resilience and emphasizes a need to implement resiliency programmes as a part of school curricula |
| River-Duval et al. 2011 (15) Mauritius | Quantitative | Resourceful Adolescent Program (RAPA) | RCT | N= 160 (from 2 single-sex secondary schools, age 12-16yrs. CG: waitlist  Six months follow-up | Universal depression prevention programme | - Significant improvement in depressive symptoms in form of hopelessness, self-esteem as well as coping skills - Improvements in self-esteem & coping skills found stable at 3 months FU. | - Showed effectiveness of the universal depression prevention programme in low-resource setting |
| Mueller et al, 2011 (16) South Africa | Quantitative | “Make a Difference (MAD) about Art” | Quasi-experimental | N= 297 youth age 8-18yrs. in one school | Community-based art Therapy Intervention | - Significant programme effect on self-efficacy scores - No improvement on other scores | - Community-based intervention showing a better utility from public health perspective |
| Kumakech et al. 2009 (17) Uganda | Quantitative | Peer-support group intervention for AIDS orphans | Cluster RCT | N= 326 children age 10-15yrs. from 20 school | Peer-support group intervention | Significant reduction in anxiety, depression and anger scores | - Peer support intervention |
| Jordans et al. 2010 (18) Nepal | Quantitative | Classroom based psychosocial intervention (CBI) | Cluster RCT | N= 325 students’ age  11-14yrs. from 8 schools Waitlist control | MH Promotion | - Showed a reduction in general psychological difficulties & aggression in boys - Increased pro-social behaviour in girls & a significant increase in sense of hope for older children | - Delivered by para-professionals |
| Khamis et al 2004, (19) Palestine | Quantitative | Classroom –Based psychosocial Intervention (CBI) | RCT | N= 664:  406 children aged 6-11yrs.  258 adolescents, age 13-16yrs.  Waitlist control | Selective prevention | - IG had significantly better attribution style, reduced level of self-blame, a higher perceived credibility, Increased inter-personal trust as well as improved communication skill - There was a reduction in hyperactivity, emotional symptoms, conduct problems, peer problems, hyperactivity significantly in adolescent - CBI had higher positive effect on adolescent girls than boys | - Importance of tapping inherent potential of the youth to develop resilience in conflict-affected areas and a replicable model for other trauma-prone areas. |
| Ager et al. 2011 (20) Uganda | Quantitative | Psychosocial Structured Activities (PSSA) intervention for displaced children aged 7-12 years in primary schools | Quasi experimental | N= 403 primary school students (mean age 10.23 yrs.), from 12 school) and  12 month follow up | MH Promotion | - Significant improvement in wellbeing of participants measured by parents & children - Girls showed more progress than boys - Older children showed more progress than younger children. | - School based multi-phased approach which can be replicated in other parts of the world |
| Quota et al. 2012 (21) Gaza, Palestinje | Quantitative | Teaching Recovery Techniques (TRT) intervention for war affected children.  Aimed at creating safety & feelings of mastery, & incorporates trauma-related psychoeducation, CBT methods, coping training. | RCT  16 sessions implemented over 4 weeks after school (2 weekly 2-hour sessions), implemented by psychologists | N= 722 children age 10-13yrs. from four schools assigned to intervention & control group  Six months follow up | Selective prevention | - Significant reduction in clinically significant Post-Traumatic Stress syndrome (PTSS) at post intervention. - Girls benefited from intervention in clinically significant PTSS if they showed low peri-traumatic dissociation | - Emphasizes on creating safety and feeling of mastery |
| Karam et al, 2008 (22) Lebanen | Quantitative | Classroom-based group intervention for children exposed to war  Aimed at reducing rates of major depressive ds., separation anxiety ds. &Post-traumatic stress disorder | Quasi-experimental  structured activities | N= 209 students  (Mean age 11.7 yrs.) from six schools Matched control group did not receive | Selective prevention | - Post-war MDD, SAD & PTSD scores were found to be associated with pre-war SAD & PTSD scores, family violence parameters, financial problems & witnessing of war events | - Intervention delivered by teachers |
| Lange-Nielsen et al. 2012 (23)  Gaza | Quantitative | Writing for Recover (WfR) intervention | RCT | N=139 adolescents age 12-17yrs. fr. 6 schools  CG: Waitlist 4-5month follow up | Aimed at improvement in PTSD & Depressive symptoms | - Significant improvement in rates of PTSD symptoms in both groups - Significant increase in IGs’ depression symptoms Significant decline in depression symptoms | - Innovative classroom-based, less stigmatizing, cost effective intervention. |
| Loughry et al, 2006 (24) Palestine  (Gaze & West Bank) | Quantitative | Child focused intervention for children living in conflict areas.  Aimed at providing structured activities to support the resilience in war-affected children Intervention also involved parents | Quasi experimental | N=400 children & adolescents | Selective  prevention | - Significant improvement in total problem scores, externalizing problem scorers, and internalizing problem scores in IG | - Involved parents as partners |
| Balaji et al., 2011 (25) Goa | Quantitative | Population based intervention to promote youth health.  Intervention implemented over 12months & consisted of 3 main components (i) Peer Education (ii) Teacher Training (iii) Health information Intervention, implemented by intervention team which consisted of social worker, two psychologist & three peer educators | Exploratory Controlled evaluation study | N= 1803 students from two urban & rural communities Control communities was wait- listed,  18 months follow up | MH promotion | - The intervention led to a significant decrease in probable depression scores (rural & urban) & lower levels of suicidality (urban) - There was an increase in self-confidence, leadership ability, stress mgt, conflict resolution, anger management & improved student-teacher relationship in peer leaders - Following intervention there was a significant change in attitudes about reproductive & sexual health (rural & urban), improvement in perpetration of physical violence (rural & urban) & substance abuse (urban) - There were significantly fewer menstrual complaints, more help-seeking for reproductive & sexual health related problems. | - Community as potential partner in planning. - Community peer education is a feasible option. |
| Vasquez et al. 2010 (26)  Honduras | Quantitative | “Familias Fuertas” (FF)  activity based sessions; local nurses trained as FF facilitators | Quasi-experimental design | N=41 parent-adolescent pairs control received informational brochures 12 months in US | Aimed at promoting consistent discipline, parental monitoring & positive communication patterns | - IG reported improvements in positive parenting behaviours as well as positive perceptions among parents about their family relationships and self-esteem of parents | - Evidence based program for family strengthening |
| Brady et al. 2007 (27)  Egypt | Quantitative | Ishraq Programme  For out of school adolescent girls age 13-15.  A Multi-dimensional  community based program aimed at improving girls’ life skills, recreational opportunities, health knowledge & attitudes & mobility & civic participation | Quasi-experimental  Girls met four times a week for 30 months in groups of 25  Programme implemented by ‘Promoters’ – in form of young local women (age 17–25) trained in their key role | N= 587 adolescent girls from four villages in Upper Egypt | MH promotion | - There was an improvement in social participation. Girls in the programme were more likely- to know about key health & rights issues - Full scale participation in program showed greater increase in academic skills - There was a strong association between desire to delay marriage & participation in Ishraq | - Utilizes local family support system |
| Jewkes et al., 2008 (28) South Africa | Quantitative | Stepping Stones,  The Collaborative HIV Adolescent MH Programme South Africa.  Programme aimed at improving sexual & emotional health by developing strong, more equal relationships | Cluster RCT  Programme delivered to single sex groups. Programme lasts 50 hours over 6–8 weeks | N= 2776 men & women age 15-26 years Two-year follow-up | Selective prevention | - There were reduced levels of depression reported in men at 24 month follow up. - Significant reduction in physical & sexual partner violence (two year follow up), problem drinking (one year follow up) and number of HSV-2 infections over 2 years in males | - Highlighted utility of multi-dimensional intervention for MH and wellbeing promotion. |
| Bell et al. 2008 (29) South Africa | Quantitative | CHAMPSA.  HIV prevention programme aimed to strengthen family relationships as well as target peer influences | RCT  10 (90 minute) sessions delivered by community caregivers over 10 weekends to families | N= 478 families rearing 579 children,  Control received existing school-based HIV prevention curriculum | MH promotion programme | - Significant improvements in caregivers’ communication skills, monitoring of children and social primary networks | - Unique in terms of focussing on caregiver’s attributes. |
| Ssewamala  et al., 2009a, 2009b, 2010, 2012 (30–33) Uganda | Quantitative | SUUBI-economic empowerment intervention by providing (i) 1–2 hrs workshops on asset building & future planning (ii) monthly mentorship program for adolescents with peer mentors on life options (iii) child development account for paying for secondary schooling, vocational training &/or family small business | RCT | N= 267 children from Grade 7 in 15 primary schools  Control group received usual care for orphaned children Ten month follow up | MH promotion | - There was a significant increase in self-esteem at 10 months post intervention, decrease in depression, increase in academic performance - A reduction in sexual risk-taking intentions and an increase in self rated physical health functioning | - Emphasized role of the multi-dimensional intervention in MH promotion and prevention. |
| Oregta et al, 2019 (34) | Editorial | - The Lancet Commission on Dementia Prevention, Intervention, & Care: a call for action - “New Life course model” of dementia prevention: nine modifiable risk factors & their potential effect in reducing individuals’ risk of dementia, | It summarizes the best available evidence to prevent & intervene for dementia. | NA | MH promotion & prevention | - Most promising intervention targets were increasing education in early life, increasing physical activity & social engagement, reducing smoking, treating hypertension, diabetes, &hearing impairment - The new model found that more than a third of dementia cases are potentially preventable. - Collectively, all nine factors account for about 35% of the population dementia risk, out of which about 20% reduction can be achieved | - Large proportion of dementia are preventable as highlighted by life course model - Interventions directed not only at people with dementia but also to their families are available & should be routinely provided to them |
| Ngandu et al, 2015, (35) Finland | Double blind RCT | - The Finnish Geriatric Intervention Study to Prevent Cognitive Impairment &Disability (FINGER) - Four intensive lifestyle-based strategies (diet, exercise, cognitive training, & vascular management) | Study compared cognition in the intervention group versus controls who received general health advice. | People with 60 – 77yrs. of age & at high risk of dementia,   599 in intervention group & 599 in control group | MH prevention | - Participants in the intervention group showed a mean improvement versus the control group in a composite measure of cognition on executive function & processing speed, but not memory. | - Multidomain intervention could improve or maintain cognitive functioning in at-risk elderly people from the general population |
| Richard et al, 2009, (36)  Charante et al, 2016, (37)  Netherlands | 6-year multi-domain, nurse-administered, open-label, cluster RCT | - Prevention of dementia by intensive vascular care (PreDIVA): a cluster-randomized trial in progress - To assess whether nurse-led intensive vascular care in primary care decreases the incidence of dementia & reduces disability - Secondary outcome parameters are mortality, incidence of vascular events, and cognitive functioning. | Aimed to reduce vascular risk factors to prevent dementia - intensive vascular care comprises treatment of hypertension, diabetes, stimulating physical exercise, & individually tailored lifestyle advices & supported by motivational interviews. | 3526 participants aged 70–78 yrs. from general practice. | MH prevention | - Initial finding: nurse-led, multidomain intervention did not result in a reduced incidence of all-cause dementia in an unselected population of older people - However, this intervention in fact led to reduction in the risk of dementia (4% in intervention group developed dementia vs 7% in control) (38) | - Highlights importance of targeted interventions |
| Forette et al, 2002, (39) | Quantitative | The prevention of dementia with antihypertensive treatment: new evidence from the Systolic Hypertension in Europe (Syst-Eur) study | DB placebo-controlled trial | 2902 patients | MH prevention | - Reduction in the incidence of dementia by more than 50% by antihypertensive treatment with nitrendipine as first line | - Early management of the risk factors of dementia such as hypertension is an effective primary prevention for dementia |
| Hamer et al , 2009 (40) | Quantitative | Physical activity & risk of neurodegenerative disease: systematic review of prospective evidence | Systematic review on prospective epidemiological studies | 163797 non-demented  participants at baseline with 3219 cases at follow-up | MH prevention | - Inverse association between physical activity & risk of dementia. | - A primordial prevention |
| Dix et al, 2012, (41) Australia | Quantitative | Implementation quality of whole-school MH promotion & students’ academic performance  **Kids Matter** -  an Australian MH intervention initiative designed to assist & include all members of the school community through four components (1) positive school community, (2) social & emotional learning for students, (3) parenting support & education, & (4) early intervention for students experiencing MH difficulties | Quasi-experimental | A random stratified sample of up to 76 students in each of 100 schools | Mental health promotion, prevention & early intervention | - KidsMatter implementation is positively associated with student academic performance | - To strengthen the claim that thequality of implementation of MH initiatives such as KidsMatter improves student socio-emotional   competencies &, academic performance. |

*Quantitative/Qualitative/Mixed method, DB: double blind, IG: intervention group, CG: control group, MH: Mental Health**,** MDD- Major Depressive Disorder, NA: not applicable, PTSD-Post traumatic Stress Disorder, SAD- Separation Anxiety Disorder, RCT: Randomized Controlled Trial

References -

1. Mishara BL, Ystgaard M. Effectiveness of a mental health promotion program to improve coping skills in young children: Zippy’s Friends. Early Child Res Q. 2006 Jan 1;21(1):110–23.

2. Clarke AM, Bunting B, Barry MM. Evaluating the implementation of a school-based emotional well-being programme: a cluster randomized controlled trial of Zippy’s Friends for children in disadvantaged primary schools. Health Educ Res. 2014 Oct;29(5):786–98.

3. Dufour S, Denoncourt J, Mishara BL. Improving Children’s Adaptation: New Evidence Regarding the Effectiveness of Zippy’s Friends, a School Mental Health Promotion Program. Adv Sch Ment Health Promot. 2011 Jan 1;4(3):18–28.

4. Holen S, Waaktaar T, Lervåg A, Ystgaard M. The effectiveness of a universal school-based programme on coping and mental health: a randomised, controlled study of Zippy’s Friends. Educ Psychol. 2012 Aug 1;32(5):657–77.

5. Clarke AM, Sixsmith J, Barry MM. Evaluating the implementation of an emotional wellbeing programme for primary school children using participatory approaches. Health Educ J. 2015 Sep 1;74(5):578–93.

6. Malti T, Schwartz SEO, Liu CH, Noam GG. Program evaluation: Relationships as key to student development. New Dir Youth Dev. 2008;2008(120):151–77.

7. Nielsen L, Meilstrup C, Nelausen MK, Koushede V, Holstein BE. Promotion of social and emotional competence: Experiences from a mental health intervention applying a whole school approach. Health Educ. 2015 Jan 1;115(3/4):339–56.

8. Caldarella P, Christensen L, Kramer TJ, Kronmiller K. Promoting Social and Emotional Learning in Second Grade Students: A Study of the Strong Start Curriculum. Early Child Educ J. 2009 Aug;37(1):51–6.

9. Yamamoto T, Matsumoto Y, Bernard ME. Effects of the cognitive-behavioral you can do it! Education program on the resilience of Japanese elementary school students: a preliminary investigation. Int J Educ Res. 2017;86:50–8.

10. Srikala B, Kishore KKV. Empowering adolescents with life skills education in schools – School mental health program: Does it work? Indian J Psychiatry. 2010;52(4):344–9.

11. Bonhauser M, Fernandez G, Püschel K, Yañez F, Montero J, Thompson B, et al. Improving physical fitness and emotional well-being in adolescents of low socioeconomic status in Chile: results of a school-based controlled trial. Health Promot Int. 2005 Jun;20(2):113–22.

12. Smith EA, Palen LA, Caldwell LL, Flisher AJ, Graham JW, Mathews C, et al. Substance use and sexual risk prevention in Cape Town, South Africa: An evaluation of the HealthWise program. Prev Sci. 2008 Dec;9(4):311–21.

13. Caldwell LL, Patrick ME, Smith EA, Palen LA, Wegner L. Influencing Adolescent Leisure Motivation: Intervention Effects of HealthWise South Africa. J Leis Res. 2010;42(2):203–20.

14. de Villiers M, van den Berg H. The implementation and evaluation of a resiliency programme for children. South Afr J Psychol. 2012;42(1):93–102.

15. Rivet-Duval E, Heriot S, Hunt C. Preventing Adolescent Depression in Mauritius: A Universal School-Based Program. Child Adolesc Ment Health. 2011;16(2):86–91.

16. Mueller J, Alie C, Jonas B, Brown E, Sherr L. A quasi-experimental evaluation of a community-based art therapy intervention exploring the psychosocial health of children affected by HIV in South Africa. Trop Med Int Health TM IH. 2011 Jan;16(1):57–66.

17. Kumakech E, Cantor-Graae E, Maling S, Bajunirwe F. Peer-group support intervention improves the psychosocial well-being of AIDS orphans: cluster randomized trial. Soc Sci Med 1982. 2009 Mar;68(6):1038–43.

18. Jordans MJD, Komproe IH, Tol WA, Kohrt BA, Luitel NP, Macy RD, et al. Evaluation of a classroom-based psychosocial intervention in conflict-affected Nepal: a cluster randomized controlled trial. J Child Psychol Psychiatry. 2010 Jul;51(7):818–26.

19. Coignez V. The Impact of the Classroom/Community/Camp-Based Intervention (CBI®) Program on Palestinian Children. 2004.

20. Ager A, Akesson B, Stark L, Flouri E, Okot B, McCollister F, et al. The impact of the school-based Psychosocial Structured Activities (PSSA) program on conflict-affected children in northern Uganda. J Child Psychol Psychiatry. 2011;52(11):1124–33.

21. Qouta SR, Palosaari E, Diab M, Punamäki RL. Intervention effectiveness among war-affected children: a cluster randomized controlled trial on improving mental health. J Trauma Stress. 2012 Jun;25(3):288–98.

22. Karam EG, Fayyad J, Nasser Karam A, Cordahi Tabet C, Melhem N, Mneimneh Z, et al. Effectiveness and specificity of a classroom-based group intervention in children and adolescents exposed to war in Lebanon. World Psychiatry Off J World Psychiatr Assoc WPA. 2008;7(2):103–9.

23. Lange-Nielsen II, Kolltveit S, Thabet AAM, Dyregrov A, Pallesen S, Johnsen TB, et al. Short-Term Effects of a Writing Intervention Among Adolescents in Gaza. J Loss Trauma. 2012 Sep 1;17(5):403–22.

24. Loughry M, Ager A, Flouri E, Khamis V, Afana AH, Qouta S. The impact of structured activities among Palestinian children in a time of conflict. J Child Psychol Psychiatry. 2006 Dec;47(12):1211–8.

25. Balaji M, Andrews T, Andrew G, Patel V. The Acceptability, Feasibility, and Effectiveness of a Population-based Intervention to Promote Youth Health: An Exploratory Study in Goa, India. J Adolesc Health Off Publ Soc Adolesc Med. 2011 May;48(5):453–60.

26. Vasquez M, Meza L, Almandarez O, Santos A, Matute RC, Canaca LD, et al. Evaluation of a strengthening families (Familias Fuertes) intervention for parents and adolescents in Honduras. Nurs Res South Online J. 2010;10(3):e1–25.

27. Brady M, Assaad R, Ibrahim B, Salem A, Salem R, Zibani N. Providing new opportunities to adolescent girls in socially conservative settings: The Ishraq program in rural Upper Egypt—full report. Poverty Gend Youth [Internet]. 2007 Jan 1; Available from: https://knowledgecommons.popcouncil.org/departments_sbsr-pgy/226

28. Jewkes R, Nduna M, Levin J, Jama N, Dunkle K, Puren A, et al. Impact of Stepping Stones on incidence of HIV and HSV-2 and sexual behaviour in rural South Africa: cluster randomised controlled trial. BMJ. 2008 Aug 7;337:a506.

29. Bell CC, Bhana A, Petersen I, McKay MM, Gibbons R, Bannon W, et al. Building Protective Factors to Offset Sexually Risky Behaviors among Black Youths. J Natl Med Assoc. 2008 Aug;100(8):936–44.

30. Ssewamala FM, Ismayilova L. Integrating Children’s Savings Accounts in the Care and Support of Orphaned Adolescents in Rural Uganda. Soc Serv Rev. 2009 Sep 1;83(3):453–72.

31. Ssewamala FM, Han CK, Neilands TB. Asset Ownership and Health and Mental Health Functioning Among AIDS-Orphaned Adolescents: Findings From a Randomized Clinical Trial in Rural Uganda. Soc Sci Med 1982. 2009 Jul;69(2):191.

32. Ssewamala FM, Karimli L, Chang-Keun H, Ismayilova L. Social Capital, Savings, and Educational Performance of Orphaned Adolescents in Sub-Saharan Africa. Child Youth Serv Rev. 2010 Dec 1;32(12):1704–10.

33. Ssewamala FM, Neilands TB, Waldfogel J, Ismayilova L. The impact of a comprehensive microfinance intervention on depression levels of AIDS-orphaned children in Uganda. J Adolesc Health Off Publ Soc Adolesc Med. 2012 Apr;50(4):346–52.

34. The Lancet Commission on Dementia Prevention, Intervention, and Care: a call for action | Irish Journal of Psychological Medicine | Cambridge Core [Internet]. [cited 2022 May 21]. Available from: https://www.cambridge.org/core/journals/irish-journal-of-psychological-medicine/article/lancet-commission-on-dementia-prevention-intervention-and-care-a-call-for-action/3E7ED3B3B08161D9FC2B75FD8268703D

35. A 2 year multidomain intervention of diet, exercise, cognitive training, and vascular risk monitoring versus control to prevent cognitive decline in at-risk elderly people (FINGER): a randomised controlled trial - PubMed [Internet]. [cited 2022 May 21]. Available from: https://pubmed.ncbi.nlm.nih.gov/25771249/

36. Richard E, Van den Heuvel E, Moll van Charante EP, Achthoven L, Vermeulen M, Bindels PJ, et al. Prevention of dementia by intensive vascular care (PreDIVA): a cluster-randomized trial in progress. Alzheimer Dis Assoc Disord. 2009 Sep;23(3):198–204.

37. Moll van Charante EP, Richard E, Eurelings LS, van Dalen JW, Ligthart SA, van Bussel EF, et al. Effectiveness of a 6-year multidomain vascular care intervention to prevent dementia (preDIVA): a cluster-randomised controlled trial. Lancet Lond Engl. 2016 Aug 20;388(10046):797–805.

38. Schneider LS. Reduce vascular risk to prevent dementia? The Lancet. 2016 Aug 20;388(10046):738–40.

39. Forette F, Seux ML, Staessen JA, Thijs L, Babarskiene MR, Babeanu S, et al. The prevention of dementia with antihypertensive treatment: new evidence from the Systolic Hypertension in Europe (Syst-Eur) study. Arch Intern Med. 2002 Oct 14;162(18):2046–52.

40. Hamer M, Chida Y. Physical activity and risk of neurodegenerative disease: a systematic review of prospective evidence. Psychol Med. 2009 Jan;39(1):3–11.

41. Dix KL, Slee PT, Lawson MJ, Keeves JP. Implementation quality of whole-school mental health promotion and students’ academic performance. Child Adolesc Ment Health. 2012 Feb;17(1):45–51.
