# Supplementary material for: Mental Health Prevention and Promotion—A Narrative Review
Source: Front Psychiatry. 2022 Jul 26;13:898009. doi: 10.3389/fpsyt.2022.898009 (PMC9360426; doi:10.3389/fpsyt.2022.898009)
Supplement: Supplementary file 2 [file Data_Sheet_1.docx]

**Search terms used and data extracted through various databases**

mental health promotion OR protection OR prevention OR mitigation

Filters:

2000 - 2019

Review articles

Original articles: 2016-2022

1. **Google scholar -** (allintitle: mental health promotion OR protection OR prevention OR mitigation)

Filters

2000 - 2019

**review articles - 225**

1. **Pubmed -** 302 (("mental health"[MeSH Major Topic]) AND ((promotion [Title]) OR (protection [Title]) OR (prevention[Title]) OR (mitigation[Title]))

Filters

2000 - 2019

review and systematic review articles

- **59 results**

1. **Cochrane** -

Filter 01/01/200 to 31/12/2019

## “Mental health in Record Title AND promotion in Record Title OR protection in Record Title OR prevention in Record Title OR mitigation in Record Title - with Cochrane Library publication date Between Jan 2000 and Dec 2019, in Cochrane Reviews (Word variations have been searched)”

Filter - **mental health 36**
